# Conjugative type IV secretion systems enable bacterial antagonism that operates independently of plasmid transfer

**DOI:** 10.1038/s42003-024-06192-8

**Published:** 2024-04-25

**Authors:** Lois Gordils-Valentin, Huanrong Ouyang, Liangyu Qian, Joshua Hong, Xuejun Zhu

**Affiliations:** 1 Department of Chemical Engineering, Texas A&M University, College Station, 77843 TX US; 2 Interdisciplinary Graduate Program in Genetics & Genomics, Texas A&M University, College Station, 77843 TX US; 3 Department of Biology, Texas A&M University, College Station, 77843 TX US

**Keywords:** Bacterial genetics, Bacterial synthetic biology

## Abstract

Bacterial cooperation and antagonism mediated by secretion systems are among the ways in which bacteria interact with one another. Here we report the discovery of an antagonistic property of a type IV secretion system (T4SS) sourced from a conjugative plasmid, RP4, using engineering approaches. We scrutinized the genetic determinants and suggested that this antagonistic activity is independent of molecular cargos, while we also elucidated the resistance genes. We further showed that a range of Gram-negative bacteria and a mixed bacterial population can be eliminated by this T4SS-dependent antagonism. Finally, we showed that such an antagonistic property is not limited to T4SS sourced from RP4, rather it can also be observed in a T4SS originated from another conjugative plasmid, namely R388. Our results are the first demonstration of conjugative T4SS-dependent antagonism between Gram-negative bacteria on the genetic level and provide the foundation for future mechanistic studies.

## Abstract (web summary)

The T4SS of RP4, a well-known conjugative plasmid in Gram-negative bacteria, displays a lethal phenotype that is independent of DNA transfer or translocated molecules and is capable of eliminating a range of Gram-negative bacterial species.

## Introduction

Microbial populations are a dynamic, diverse, and critically important component of any ecological system^1–3^. The ubiquity of microbes cannot be understated, as they are present in a myriad of environments, ranging from bodies of water to soils, to even plants and animal systems^3–5^. Regardless of the environmental setting, however, bacterial interaction plays an essential part in shaping entire communities, as microbes can either cooperate amongst themselves or antagonize each other^6–12^.

Strategies for bacterial antagonism can either be non-contact-dependent or contact-dependent. For non-contact dependent, bacteria can: (1) produce specialized metabolites for the inhibition of competitor cells (e.g., antibiotic production by *Streptomyces*), (2) deplete essential nutrients (e.g., siderophores sequestering iron), or (3) produce enzymes that interfere with competitor cell’s life cycle (e.g., Esp production by *Staphylococcus epidermidis* blocks *Staphylococcus aureus* ability to form biofilms)^10–14^. On the other hand, contact-dependent strategies can: (1) deliver toxins to opposing cell membrane receptors (e.g., the *E. coli* EC93 CDI system) or (2) use specialized secretion systems to directly inject toxins inside competing bacteria (e.g., type VI secretion system (T6SS) or type IV secretion systems (T4SSs)^12,15–18^. Moreover, the antagonistic capabilities of bacterial secretion systems have piqued the interest of researchers looking to exploit them for antibacterial technologies, such as the programmable and targeted killing of cells in mixed populations using a T6SS, the use of conjugation to deliver lethal cargo that encodes antimicrobial molecules (e.g., CRISPR-Cas-based nucleases and toxin-intein systems) with great efficacy, as well as a recently uncovered type IVB secretion system (T4BSS) on *Pseudomonas putida* that can inject toxins into competitor cells in a contact-dependent manner with potential applications in biocontrol^19–22^. These advances highlight the versatility of secretion systems, either natural or synthetic, that bacteria utilize, to compete against other members of their community.

A major family of secretion systems are the T4SSs involved in bacterial conjugation-dependent horizontal gene transfer. T4SS-mediated conjugation is a well-known mechanism for bacterial cooperation. For example, conjugation can disseminate antibiotic resistance genes within bacterial communities to allow them to thrive under antibiotic stress. It can also contribute to the spread of hypervirulence plasmids to allow the recipient population to colonize bodily tissues^23–26^. While the role of conjugative T4SSs in cooperative interactions has been well-studied, their potential in bacterial antagonism has been overlooked for decades. Although there have been reports about bacteria-killing T4SS, such as *Xanthomonas*, *Lysobacter, Stenotrophomonas*, and even *P. putida* delivering toxic payloads to target cells, it should be noted that these rely on translocated effectors and are not strictly conjugative in nature^17,18,22,27–29^. On the other hand, recent work leveraging conjugative T4SS has shown that conjugative T4SSs can enable interbacterial antagonism by transferring DNA-based cargo into target cells^19–21,30^. However, all of the examples mentioned, whether natural or artificial, are dependent on the effective delivery and/or expression of some type of molecular cargo. Another overlooked example of antagonism mediated by conjugative systems was reported in the 1950’s^31^. Characterized by a steep decline in viable recipient cells after exposure to an excessive amount of high-frequency recombination (Hfr) donor cells, this deadly event was dubbed lethal zygosis. The features of the lethal event based on the F system include (a) the dependence on close cell-to-cell contact (b) the formation of a mating pair using the conjugative apparatus, (c) independence of horizontal gene transfer as indicated by the failure of detecting viable F^-^ plasmid after the exposure to F^+^ plasmid when the horizontal gene transfer was blocked by nalidixic acid^32^, (d) the collateral transfer of genetic material that prevents recipient cell death as seen by the few surviving transconjugants^32^. These characteristics of this lethal effect suggest that the mating pair formation system of F enables bacterial antagonism in a manner independent of horizontal gene transfer. Of note is that this phenomenon alludes only to F-based systems and, to the best of our knowledge, has not been shown on any other conjugative platform. In addition, genetic determinants of the lethal phenomenon observed in the F system remain unclear to this day^31–35^. Nevertheless, because almost all conjugative plasmids encode the mating pair formation system (referred to as T4SS) to enable close cell-to-cell contact, we hypothesize that conjugative T4SS may harbor a potential for enabling bacterial antagonism.

Here, we have expanded upon this phenomenon by uncovering the antagonistic property of T4SS originated from conjugative plasmid RP4 using engineering approaches. RP4 (also known as RK2) is a well-studied plasmid that belongs to a different incompatibility group (IncP) than the F system (IncF), but the antagonism by its T4SS has gone unnoticed since the first isolation of plasmid RP4 in the 1960s^36–41^. Our engineering efforts on this conjugative system imply that the antagonistic activity of T4SS from RP4 is, as far as we know, independent of molecular cargo being delivered into targeted cells. Moreover, we scrutinized the genes conferring resistance to this T4SS-dependent antagonism and showed its dependency on cell-to-cell contact. Furthermore, the lethal phenotype was observed to be effective against Gram-negative bacteria in both mono- and mixed-culture settings. Finally, we demonstrated that such an antagonistic property is not limited to T4SS sourced from RP4, rather it can also be observed in a T4SS derived from another conjugative plasmid belonging to another incompatibility group (i.e., IncW), namely R388^42–44^. Our results are the first demonstration of conjugative T4SS-dependent antagonism between Gram-negative bacteria on a genetic level and provide the foundation for future mechanistic studies.

## Results

### An RP4 derivative incapable of transfer endows the host cell with antagonistic properties

**Fig. 1:**
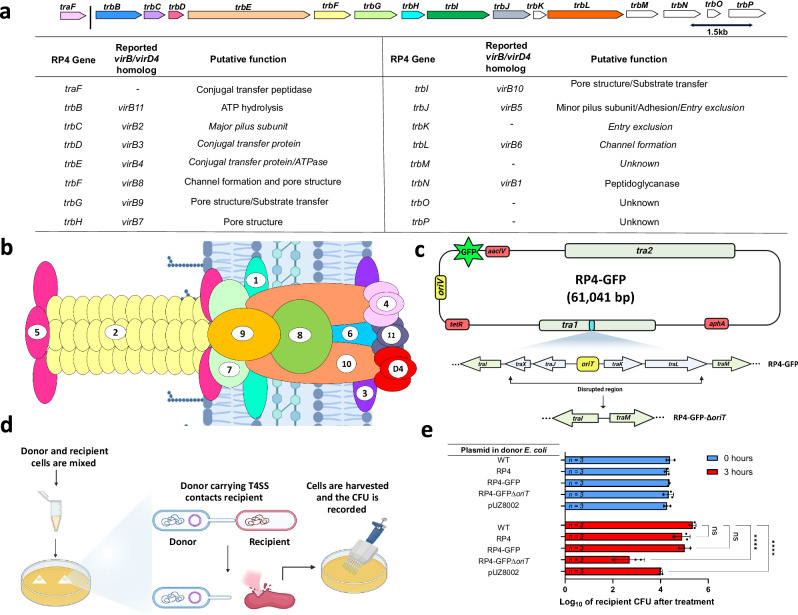
Antimicrobial potential of plasmid RP4. **a** Schematic that shows the *tra2* region and *traF* from *tra1* operon of plasmid RP4. Colored blocks signify that they have been reported as part of T4SS. The table beneath summarizes these genes and their respective homologues reported in the *virB/virD4* T4SS and the putative function. **b** Schematic of the *virB/D* T4SS archetype from *Agrobacterium tumefaciens*. The figure is modified from S. Ananiadou, D. et al. ^103^. Numbers correspond to the respective *vir* gene (e.g., #5 is *virB5*). (created with BioRender.com). **c** Schematic of the RP4-GFP-∆*oriT* plasmid alongside modifications done to the *oriT* and genes *traXJKL* inside the *tra1* region using lambda red recombineering. Antibiotic resistance genes are colored red (*aacIV* = apramycin; *tetR* = tetracyline; *aphA* kanamycin). (created with BioRender.com). **d** An overview of the experimental approach (created with BioRender.com). **e** Recipient *E. coli* DA32838 CFU after 0 and 3 hours of exposure to an *E. coli* NEB® 10-beta donor equipped with RP4, RP4-GFP, RP4-GFP-∆*oriT* or pUZ8002. Plasmidless *E. coli* NEB® 10-beta were used as control. The initial donor CFU was approximately set to the order of 10^7^. The raw CFU of the recipient was first log_10_ transformed, and the data shown is the mean of the log_10_ transformed data, with the sample size shown inside the corresponding bar. Error bars represent the standard deviation of the log_10_ transformed data. *P* values were obtained by doing a two-way RM ANOVA followed by Dunnett’s multiple comparisons test. **** *P* < 0.0001; ns, not significant.

To evaluate the antagonistic potential of conjugative T4SSs, we chose RP4 as our model system. RP4 is a well-known conjugative plasmid that expresses all the requisite genes for a T4SS, which is ancestrally related to the T4SS found in *Agrobacterium tumefaciens* (Figs. 1a and b)^37,39,45–49^. This machine is a complex macromolecular secretion system, that grants RP4 the ability to self-transfer across a broad range of Gram-negative bacteria^37,39,41,45–47^. Since RP4 encodes both T4SS and DNA-processing machinery for self-transmissibility, an indispensable step to probe the antagonistic property of T4SS is to decouple it from the self-transfer function. To this end, we first incorporated GFP onto RP4, denoted as RP4-GFP, followed by the disruption of the DNA-processing machinery^39,50^ (*oriT* and *traLKJX*) to generate RP4-GPF-*ΔoriT* (Fig. 1c). The incorporation of GFP onto the plasmids is to better visualize plasmid transfer based on fluorescence. By transforming the corresponding plasmid into an *E. coli* NEB® 10-beta, we prepared four types of donors containing RP4, RP4-GFP, RP4-GFP-*ΔoriT*, and no plasmid, respectively. In addition to these, we also incorporated pUZ8002, an RP4 derivative with a deficient *oriT* commonly used in *Streptomyces* genetics^51,52^. To evaluate the donors’ antagonistic capabilities, these were incubated individually with *E. coli* DA32838 as the recipient strain (Fig. 1d)^53^. Briefly, we mixed the donor and recipient *E. coli* and spotted the mixture on cellulose acetate filters (0.45 µm pore size) placed on an agar medium. Cells were harvested at both 0 and after 3 hours of incubation. Since donor and recipient cells, and the plasmids all possess unique antibiotic markers, we quantified the colony-forming units (CFUs) of donors, recipients, and transconjugants (i.e., recipient cells that have acquired the plasmid from the donor cells due to bacterial conjugation) by plating the bacterial cell mixture on appropriate antibiotic agar media. As expected, after 3 hours of incubation, donors containing RP4-GFP-*ΔoriT* or pUZ8002 led to no transconjugants in contrast to those containing RP4-GFP or RP4 (Supplementary Fig. 1a). Strikingly, compared to the treatment with the donors containing RP4-GFP, RP4, or no plasmid, there was a ~100-fold reduction in viable recipient CFU after the exposure to the donor containing RP4-GFP-*∆oriT* (Fig. 1e). The lethal effect is not due to the overgrowth of the donor as shown by the similar CFU as the other types of donors after the 3-hour treatment (Supplementary Fig. 1b). Moreover, not only can pUZ8002 display a similar lethal phenotype (Fig. 1e) and be non-self-transmissible (Supplementary Fig. 1a), but it is also able to transfer mobilizable plasmids (i.e., pIB139-based plasmid) that carry an *oriT* (Supplementary Table 2). This result rules out the possibility that the killing observed in the RP4-GFP-*∆oriT* plasmid is due to the incorrect assembly of the T4SS caused by the elimination of *traXJKL* during the plasmid construction of RP4-GFP-*ΔoriT*. To further support this statement, we knocked out *traK* in plasmid RP4, which is a gene involved in *oriT* recognition that is essential for DNA transfer^37,40,46^. Results show that the removal of *traK* was sufficient to replicate the lethal phenotype observed in the RP4-GFP*-∆oriT* strain (Supplementary Fig. 2). Overall, these results demonstrate that the antagonistic activity is independent of plasmid transfer and are not the result of incorrect or malfunctioning T4SS. Additionally, donors containing RP4 and RP4-GFP, which were fully capable of self-transfer (Supplementary Fig. 1a), resulted in an almost negligible reduction in recipient CFU in comparison with the plasmid-free donor (Fig. 1e), suggesting that disrupting the DNA-processing machinery involved in plasmid transfer is essential for the lethal effect. A hypothesis for this phenomenon is that RP4 and RP4-GFP carry a resistance mechanism that can only be acquired by the recipient through plasmid transfer. The rest of this work is devoted to characterizing the antagonism and identifying cognate resistance genes towards it.

### The antagonistic phenotype is dependent on T4SS

**Fig. 2:**
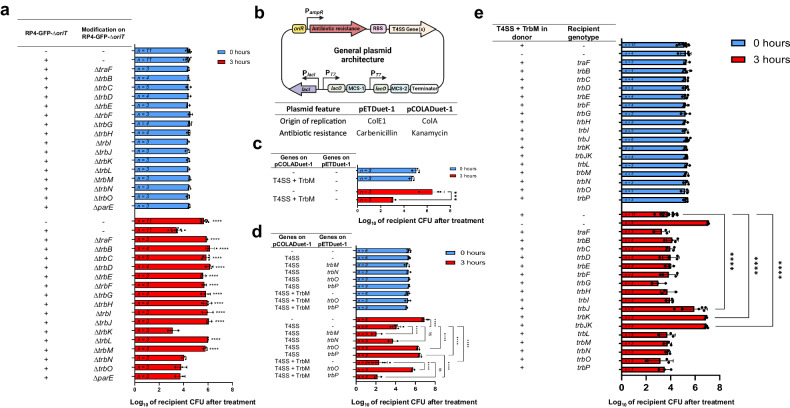
Genetic determinants behind the antagonistic phenotype. **a** The results of the knockout studies performed on the RP4-GFP-*ΔoriT*. The initial donor CFU was approximately set to the order of 10^7^. **b** General plasmid architecture used for the pCOLADuet-1 and pETDuet-1-based constructs (created with BioRender.com). **c** The results of the T4SS reconstitution of traF*/trbBCDEFGHIJKLM*. **d** The effects of expression of *trbN, trbM, trbO*, and *trbP* on the lethality of the reconstituted T4SS. **e** The resistance mechanism towards the reconstituted T4SS antimicrobial was assayed using *E. coli* DA32838 recipients carrying either an empty pETDuet-1 vector or a T4SS-associated gene. Shown is the recipient CFU after 0 and 3 hours of treatment with *E. coli* NEB® 10-beta donors equipped with either the reconstituted T4SS + TrbM or an empty pCOLADuet-1 vector. The initial donor CFU for figures **c**–**e** was approximately set to the order of 10^6^. For **a**, **c**–**e**, the raw CFU of recipients was first log_10_ transformed and the data shown is the mean of the log_10_ transformed data, with the sample size shown inside the corresponding bar. Error bars represent the standard deviation of the log_10_ transformed data. For **a**, two-way RM ANOVA were performed followed by Dunnett’s multiple comparisons test. *P* values shown belong to mutants that abolished the phenotype and were compared to the RP4-GFP-∆*oriT* control. For **c**, two-way RM ANOVA was performed followed by’Šídák’s multiple comparisons test. For **d**, two-way RM ANOVA was performed followed by Tukey’s multiple comparisons test. For **e**, two-way RM ANOVA was performed followed by Dunnett’s multiple comparisons test were performed. ***P < 0.001; ****P < 0.0001; ns, not significant.

To characterize the antagonistic phenotype displayed by RP4-GFP-*∆oriT*, we sought to determine the genetic factors behind it. With our knowledge of conjugation machinery and its ancestral relationship to T4SS^54,55^, we focused our efforts on the *tra2* operon and *traF* from the *tra1* operon of RP4. *TraF* and at least ten additional genes (i.e., *trbBCDEFGHIJL*) in the *tra2* operon are known to be essential for the assembly and function of the RP4-T4SS machinery^39^ with reported homologues on a representative T4SS (i.e., *virB/virD4* T4SS from *Agrobacterium*)^49,56,57^ (Fig. 1a). To probe their roles in promoting bacterial antagonism, we generated individual mutants of these T4SS genes on RP4-GFP-*∆oriT* by replacing the target gene with a spectinomycin resistance marker (*aadA*). These mutants were individually transformed into donors to treat recipient *E. coli* DA32838. The antimicrobial ability was abolished in all these mutants (Fig. 2a). The antagonistic phenotype of the mutants, including *∆traF, ∆trbF, I, J*, and *L*, could be restored through genetic complementation assays where the wild-type (WT) gene was re-introduced on the pETDuet-1 vector to the corresponding mutant (Supplementary Fig. 3), demonstrating the crucial roles of these genes. However, the antagonistic phenotype of other mutants (i.e., *∆trbB*, *C*, *D*, E*, G*, and *H*) could not be restored through complementation, possibly due to the polar effects caused by the inserted *aadA* marker in these mutants. We later demonstrated the crucial roles of *trbBCDEGH* using complementation assays based on the reconstitution approach as discussed in the paragraph after the next.

Moreover, we disrupted four extra genes in the *tra2* operon of RP4-GFP-*∆oriT* (i.e., *trbK*, *trbM*, *trbN*, and *trbO*) that are not known to form part of the T4SS but are in the same operon^41,45,46,58^. The *∆trbK*, *∆trbN*, and *∆trbO* mutants still possessed the antagonistic potential after removal, making them dispensable for the antibacterial effect (Fig. 2a). Interestingly, the removal of *trbM* eradicated the antagonistic capability, despite not being previously reported as an essential part of the T4SS (Fig. 2a)^37,40,41,46^. To further probe its potential function, we disrupted *trbM* from the fully-transferable RP4-GFP construct. This resulted in an abolishment of conjugative transfer that could be restored by complementing the mutant with the WT *trbM* gene on a pETDuet-1 vector, strongly suggesting that *trbM* is crucial for conjugative transfer (Supplementary Fig. 4). Aside from these and to rule out the native plasmid addiction system *parDE*^59–61^ contributing to the lethal phenotype, we disrupted the *parE* toxin gene in RP4-GFP-*∆oriT* and showed that it has no effect in the killing efficiency (Fig. 2a). Overall, these gene disruption assays demonstrate that: (1) the T4SS is indeed involved in the antimicrobial effect, (2) *trbM* plays an important role in both the observed the phenotype and conjugative transfer, and (3) the RP4 *parDE* toxin-antitoxin system does not contribute to the observed lethal effect.

To determine whether T4SS genes (namely *traF* and *trbBCDEFGHIJKL*) and *trbM* are sufficient for the antimicrobial effect, we reconstituted them under the constitutive expression by the a*mpR* promoter on the pCOLADuet-1 cloning vector (Fig. 2b). Gene *trbK* was included for cloning convenience. Here thereafter, whenever T4SS genes are mentioned, it also includes *trbK* unless stated otherwise. The co-expression of these proteins endowed the donor strain with a strong antagonistic phenotype, as demonstrated by the CFU reduction of the recipient after 3-hours of exposure compared to the control where a donor carried an empty vector was used (Fig. 2c). Moreover, to rule out *E. coli* strain-specificity contributing to the killing observed in the reconstituted system, we treated a recipient *E. coli* DA32838 strain with two additional *E. coli* donor strains (i.e., DA32838 and XL1-Blue) equipped with either the reconstituted T4SS + TrbM or an empty pCOLADuet-1 vector control. Results show that different *E. coli* donors share comparable killing activity against the recipient strain, ruling out strain-specificity as a contributing factor to the observed killing (Supplementary Fig. 5).

As mentioned previously, to further determine the essential roles of *trbBCDEGH* that failed to complement the corresponding mutants of RP4-GFP-*∆oriT* (Supplementary Fig. 3), we removed these individual genes from the reconstituted system. These knockouts abolished the antimicrobial phenotype, which could be restored by complementation with the WT gene (Supplementary Fig. 6), thus demonstrating their indispensable roles. Additionally, reports of T4SSs capable of transferring plasmids containing conjugative machinery belonging to other T4SSs suggest that crosstalk between different T4SS is possible, hinting at the possibility that components from a related T4SS could complement a knockout of its respective homologue^62,63^. To test this hypothesis, we transformed the donor strains carrying either *∆trbB* or *∆trbJK* mutant of T4SS + TrbM with their respective *virB/D4* homologues, namely *virB11* and *virB5*^48,49^. Due to difficulties in obtaining a proper *trbJ* knockout on the reconstituted T4SS + TrbM, a *trbJK* knockout was used here instead. Fortunately, the removal of *trbK* does not hamper the killing activity of either the RP4-GFP*-∆oriT* construct (Fig. 2a) or on the reconstituted T4SS (Supplementary Fig. 7a). Next, we performed complementation assays as done for the reconstitution experiments done for Supplementary Fig. 3. Results show that both *∆trbB* and *∆trbJK* were unable to be complemented by their respective *virB/D4* homologue (Supplementary Fig. 7b). This suggests that the interactions between the essential components of the RP4-T4SS are specific and have diverged greatly from the *virB/D4* T4SS.

### Non-T4SS genes can alter the antagonistic phenotype of the donor

Next, we set out to explore how non-T4SS genes within the same operon, namely *trbM*, *N*, *O*, and *P*, could affect the lethality displayed by the donor. These four genes are not known to be part of the core region necessary for plasmid transfer between bacteria, with very limited reports available regarding their function. What we do know is that *trbM* is most likely exported across the cell membrane, due to the presence of a signal peptide within its sequence^64^. Nonetheless, the general consensus is that *trbM* is a non-essential protein for intraspecific mating in *E. coli*^40,65^. This is supported by past work showing that *trbM* on plasmid R751, a homologue of *trbM* found in plasmid RP4 (76.73% amino acid identity and E-value 4e-90 using BLASTp), is not crucial for conjugative transfer to take place^66^. However, our results from our *trbM* knockout and complementation assays contradict these notions and show that it is essential for conjugative transfer in plasmid RP4 (Supplementary Fig. 4). On the other hand, *trbN* is a homologue of *virB1* in the *A. tumefaciens* system, which codes for a murein-degrading enzyme that is non-essential for conjugative transfer of DNA^49^. Regarding *trbO* and *trbP*, virtually no information is available regarding their function, with the only noteworthy report of *trbP* being that it appears to be a homologue of *traX* of the F plasmid, which is a pilin acetylase^45,67^. Nonetheless, neither *trbO* nor *trbP* have been reported to be part of or contribute to the conjugative transfer of plasmid RP4^43,45,58,68^.

Since the successful reconstitution of the lethality on the pCOLADuet-1 vector provides a more convenient and simplified cloning platform, we decided to investigate the impact of *trbM*, *N*, *O*, *P* on the lethal phenotype based on the reconstituted T4SS. We first removed *trbM* from the reconstituted system. Next, we performed experiments using a two-plasmid system in which the T4SS genes were on pCOLADuet-1 and either *trbM*, *N*, *O*, or *P* were on pETDuet-1. Surprisingly, results show that T4SS by itself, without *trbM*, is enough to kill (Fig. 2d). The addition of *trbO* or *trbP* both abolished the lethality of the donor (Fig. 2d). The addition of *trbM* into the system, either on the initial reconstituted plasmid that included *trbM* in the same operon as T4SS genes or in the complementation plasmid, enhanced the killing (Fig. 2d). Although the findings regarding *trbM* seemed inconsistent with our initial gene disruption of *trbM* on the RP4-GFP-*∆oriT* shown in Fig. 2a and Supplementary Fig. 4, in which *trbM* was shown to be essential for both lethal phenotype and conjugation, it could possibly be explained by the co-existence of T4SS genes alongside *trbO* or *trbP* on the *∆trbM* mutant of RP4-GFP-*∆oriT*. The enhancing effect of *trbM* on the lethal phenotype is possibly due to its role in strengthening intercellular contact, as previous reports on its homologues have revealed their roles in efficient conjugative DNA transfer by enhancing contact^66,69^. Unlike *trbMOP*, the addition of *trbN* had no detectable impact on the lethal effect.

Next, we investigated whether *trbM* could overcome the impact of the expression of *trbO* or *trbP* by introducing them individually on a pETDuet-1-based-plasmid together with a reconstituted pCOLADuet-1 based plasmid that contained both T4SS-genes and *trbM* into donor cells. From our assays, when compared to the recipients treated with donors containing the T4SS + TrbM, the antagonistic phenotype was preserved if both *trbM* and *trbP* were present but lost when *trbO* was expressed even though *trbM* was also in the reconstituted system (Fig. 2d). We further confirmed that the observed phenotypes were not due to the poor expression of *trbM*, *trbO* or *trbP* by using LacZ fusion assays. Specifically, the expression levels of the LacZ fused to the TrbM in the strains cotransformed with T4SS + TrbM and pETDuet, trbO, or trbP were comparable, showing that the presence of TrbO or TrbP has no discernible impact on the expression TrbM in the T4SS + TrbM construct (Supplementary Fig. 8a). Expression levels of both TrbO and TrbP complementation plasmids remained at similar levels as well in the presence of the reconstituted T4SS + TrbM construct (Supplementary Fig. 8b). However, it should be noted that our reconstitution experiments cannot exclude the possibility that other genes and regulation systems found in RP4 could also play a role in the interactions between T4SS, *trbM*, *N*, *O*, and *P*. Since the expression of *trbM* can enhance the antimicrobial activity of T4SS and even overcome the negative impact of *trbP* in the reconstituted system, we decided to include *trbM* in the donor containing the reconstituted T4SS genes hereafter to further characterize the antagonism unless otherwise stated.

### Conjugative T4SS itself encodes cognate resistance to the lethal phenotype

Having scrutinized the genetic determinants for the antagonism, the next step was to elucidate its resistance mechanism. Since RP4-T4SS is known for being tied to conjugation, we focused on membrane proteins (i.e., TrbJ and TrbK) that have been previously reported to prevent the transfer of RP4 to other cells via entry exclusion^70–72^. TrbJ, is a homologue of the VirB5 protein in the *Agrobacterium tumefaciens* T4SS, a component of the T4SS that is crucial for conjugative transfer of DNA in said microbe^73,74^. Studies have shown that VirB5 localizes to the tip of the T4SS pilus and could play a role in mediating host recognition and adhesion^73,75^. On the other hand, TrbK is a small lipoprotein that has been reported to be involved in entry exclusion. This protein is processed and localized to the cytoplasmic membrane of the cell where it exerts entry exclusion^72^. Due to the role of these proteins in blocking conjugation, we hypothesized that these proteins would also confer resistance to the antagonistic phenotype. As such we expressed them individually on pETDuet-1, as well as together, in a recipient *E. coli* DA32838 strain. For rigorousness, we also cloned other genes from the *tra2* operon, as well as *traF*, on a cloning vector. We then expressed them individually in the recipient strain and tested them for resistance. Consistent with our hypothesis and past work^70,72^, only *trbJ* and *trbK* expression on the cloning vector allowed the recipient cell to resist exposure to the donor (Fig. 2e). These results are further supported by the observation that recipient cells carrying a derivative of the reconstituted T4SS+TrbM in which the entry exclusion proteins TrbJ and TrbK were removed were susceptible to the lethality of the donor (Supplementary Fig. 9).

Moreover, we wondered if natural conjugative plasmids that contain entry exclusion genes could confer resistance against our reconstituted antimicrobial. For this, we introduced natural conjugative plasmids, RP4, R388, and R6K, individually into a recipient *E. coli* strain^76,77^. Results showed that our reconstituted antimicrobial based on RP4-T4SS was indeed hampered by RP4 that carries *trbJK*. The other conjugative plasmids tested did not increase recipient cell survival against our reconstituted antimicrobial compared to the susceptible wild-type recipient cell, suggesting that the resistance and its associated systems are specific to the T4SS that encodes it (Supplementary Fig. 10).

### The observed lethal effect is contact-dependent and is not due to host-secreted molecules

**Fig. 3:**
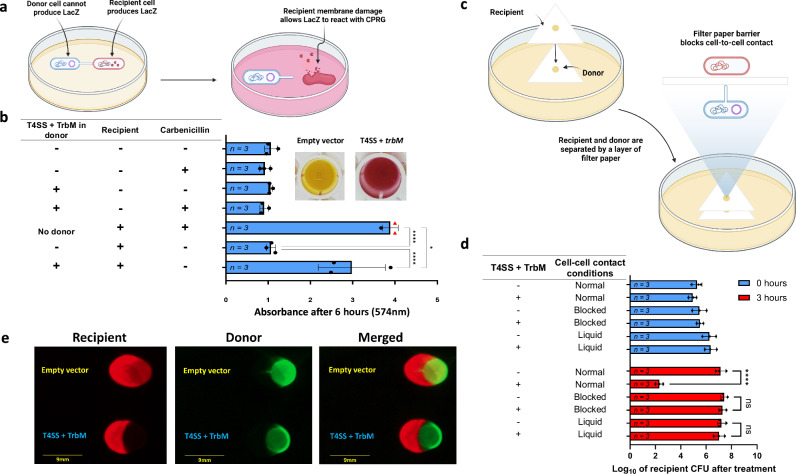
Killing is dependent on cell-to-cell contact. **a** An overview of the in situ colorimetric assay (created with BioRender.com). **b** The results of the in-situ assay at 574 nm. CPRG was added in the LB agar in each well. Carbenicillin was added into the well if shown as “+” in the table. The addition of carbenicillin in the corresponding wells was used as a control, as both donor and recipient *E. coli* strains were sensitive to carbenicillin. Red triangles in the graph indicate that the reading overflowed, so the detection limit (4.0) was used instead. Shown to the right of the graph are representative wells 16 hours after incubation in which the recipient *E. coli* DA32838 was exposed to an *E. coli* NEB® 10-beta donor containing either an empty pCOLADuet-1 vector or T4SS + TrbM on LB agar supplemented with CPRG (200 µg/mL). **c** Schematic of a modified mating experiment to demonstrate the antimicrobial’s dependency on cell-to-cell contact. Donor *E. coli* NEB® 10-beta cells are initially spotted on a piece of cellulose acetate filter paper. Afterward, a second layer is placed on top and recipient cells are spotted directly on top of where the donor was spotted (created with BioRender.com). **d** CFU of *E. coli* DA32838 recipient cells after 0 and 3 hours of exposure to donor *E. coli* NEB® 10-beta cells under modified treatment conditions. Cell-to-cell contact was promoted as usual in the samples denoted as “Normal”, while antimicrobial activity was assayed in conditions that blocked direct cell-to-cell contact (denoted as “Blocked” as shown in the upper panel) and in a liquid environment (denoted as “Liquid”). The donor strains either carried an empty pCOLADuet-1 vector or the reconstituted T4SS+TrbM. **e** CDK assay in which donor and recipient colonies were partially overlaid. The recipient strain expressed dTomato and the donor strains expressed GFP. Recipients were treated with either a donor carrying the T4SS + TrbM construct expressing GFP (labeled with “T4SS + TrbM” in the figure), or a control donor equipped with a pCOLADuet-1 vector expressing GFP (labeled with “Empty vector” in the figure). The images shown here were taken after a total of six hours of incubation (37 °C for 3 hours, followed by 30 °C for 3 hours). For the recipient strain, images were taken with an imaging system using the 515 – 545 nm excitation filter and the 568 – 617 nm emission filter. Whereas for the donor strains, images were taken using the 455–485 nm excitation filter and the 508–557 nm emission filter. Both donor and recipient strains shared the same genomic background (*E. coli* NEB® 10-beta). The unedited images, as well as the raw and the fluorescent images corresponding to Fig. 3e, are shown in Supplementary Fig. 11d,  12d, and  13b. For **b**, the raw absorbance values at 574 nm were used and the data shown is the mean, with the sample size shown inside the corresponding bar. Error bars represent the standard deviation of the absorbance values. RM one-way ANOVA was performed followed by Tukey’s multiple comparisons test. For **d**, the initial donor CFU was approximately set to the order of 10^7^. The raw CFU of the recipient was first log_10_ transformed and the data shown is the mean of the log_10_ transformed data, with the sample size shown inside the corresponding bar. Error bars represent the standard deviation of the log_10_ transformed data. Two-way RM ANOVA was performed followed by Tukey’s multiple comparisons test. *P < 0.05; ****P < 0.0001; ns, not significant.

Because the T4SS involved in conjugation works via cell-to-cell contact^54,78,79^, we wondered if the antimicrobial functions in a similar way. To answer this, we performed chlorophenol red-β-D-galactopyranoside (CPRG) assays. For these experiments, we paired our recipient *E. coli* DA32838 lab strain against a donor *E. coli* NEB 10-beta strain possessing either an empty pCOLADuet-1 vector or T4SS genes + TrbM directly on agar (no filter paper used) supplemented with CPRG (Fig. 3a). CPRG has been widely used as a colorimetric substrate for LacZ to assess membrane permeability^80^ or cell lysis in situ^81^. Since only the recipient strain has a fully functional LacZ that can be released onto the medium when its membrane is compromised, the appearance of a red color due to the LacZ-catalyzed hydrolysis of CPRG implies membrane damage and lysis of recipient cells. In comparison to the empty pCOLADuet-1 vector control, we observed a considerable color change when the recipient was treated with the donor carrying T4SS + TrbM, as noticed by the absorbance reading and the naked eye (Fig. 3b), thus suggesting that the lethal effect is associated with membrane damage or cell lysis caused by the conjugative machinery. However, this assay alone is insufficient to demonstrate a dependency on cell-to-cell contact. Therefore, we performed a modified experiment in which donor-to-recipient contact was disrupted by the addition of an extra membrane filter (0.45 µm pore size) between the strains (Fig. 3c), as well as liquid culture assays with constant shaking in which conjugation has been reported to be much less frequent due to transient cell-to-cell contact^82^. In both conditions, the phenotype was abolished (Fig. 3d). This demonstrates that the lethal phenotype is dependent on intercellular contact. To further support this conclusion, we performed contact-dependent killing (CDK) assays in which the donor and recipient strains were fluorescently tagged, and part of the recipient colony covered the donor colony. Having shown the dependency on close intercellular contact, we expect a substantial lack of fluorescent signal corresponding to the recipient (red) only in the area in which it is directly contacting the donor (green). As shown in the fluorescent images, the area occupied by the T4SS + TrbM donor is devoid of red fluorescence when compared to the empty vector control (Fig. 3e, Supplementary Figs. 11–13). This serves as further proof that the lethal phenotype is reliant on cell-to-cell contact. Additionally, the intense red fluorescence signal present in the rest of the colony rules out the possibility of the killing being the result of host-secreted molecules diffusing through the media. Nevertheless, this does not omit host-secreted molecules being delivered directly into the recipient via the T4SS as a contributor to the phenotype. To address the concern of whether the antagonistic effect is linked to chromosome-associated molecules being transported across the reconstituted T4SS and into the target strain. We used the same parent *E. coli* strain as both the donor and the recipient. The donor strain expressed the reconstituted T4SS and TrbM on a cloning vector, while the recipient strain carried an empty cloning vector expressing a different antibiotic resistance marker for strain differentiation. If there is a chromosome-associated T4SS-transferrable molecule essential for the killing effect, we would expect that the donor should also contain chromosomal genes to protect itself from being killed in the first place. Since both donor and recipient *E. coli* have the same genetic background except the plasmid carried, the recipient is expected to also have immunity and hence will not be killed by the donor. From our data, the recipient *E. coli* can still be eliminated by the donor (Supplementary Fig. 14), implying that chromosome-associated molecules being passed through T4SS, if there are any, do not contribute to recipient cell death. This, alongside the other experiments performed, supports that the mode of killing: (1) is dependent on close cell-to-cell contact, (2) does not depend on host-secreted molecules diffusing through the media or being delivered into the recipient through the T4SS^80,81^.

### The lethal effect is dependent on the number of donor cells and is not limited to *E. coli* recipients

**Fig. 4:**
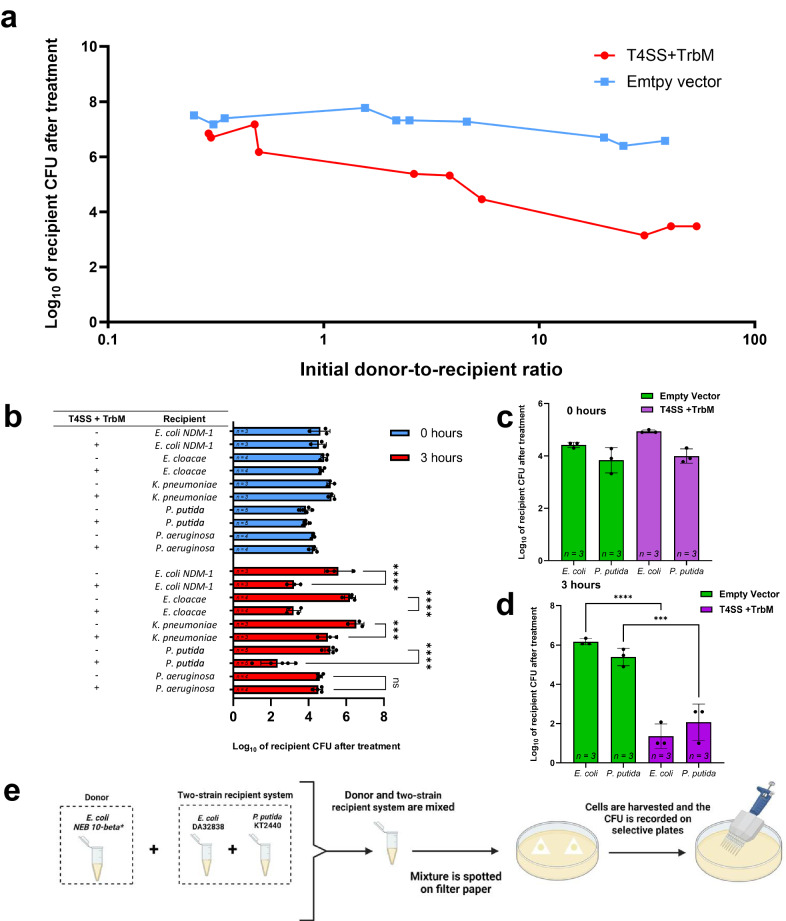
The Lethal effect is dependent on the donor-to-recipient ratio and is effective against different Gram-negative bacteria and mixed recipient populations. **a** The effect of different initial donor-to-recipient ratios on the antimicrobial effect of the T4SS + TrbM donor against the *E. coli* DA32838 recipient. The raw CFU of recipients after the 3-hour treatment was log_10_ transformed and the data shown was collected from experiments performed on four different days. Each data point corresponds to a single replicate. **b** The efficacy of the antimicrobial against *E. coli* NDM-1 (BAA-2452), *Enterobacter cloacae* (BAA-2468), *Klebsiella pneumoniae subsp. pneumoniae* (derived from ATCC BAA-2524), *Pseudomonas putida* KT2440 (ATCC 47054) and *Pseudomonas aeruginosa* PAO1-LAC (ATCC 47085). **c** Lethal effect of the donor carrying the T4SS + TrbM against mixed recipient cell population containing *E. coli* DA32838 and *P. putida* KT2440 after 0 hours of treatment. **d** Lethal effect of the donor carrying the T4SS + TrbM against mixed recipient cell population containing *E. coli* DA32838 and *P. putida* KT2440 after 3 hours of treatment. **e** Schematic depicting the experimental setup for assays performed for figures **c** and **d**. (created with BioRender.com). For figures **a**–**d**, the donor background was *E. coli* NEB® 10-beta. For figures **b**–**d**, the initial donor CFU was approximately set to the order of 10^7^. Whenever no recipient colonies were observed, the CFU detection limit of [(1 × 10^0^)/5 µL]*50 µL = 10 was used to enable the subsequent log_10_ transformation. The raw CFU of recipients was first log_10_ transformed and the data shown is the mean of the log_10_ transformed data, with the sample size shown inside the corresponding bar. Error bars represent the standard deviation of the log_10_ transformed data. For **b**, two-way RM ANOVA was performed followed by Tukey’s multiple comparisons test. For **d**, ordinary one-way ANOVA followed by Šídák’s multiple comparisons test. ***P < 0.001; ****P < 0.0001; ns, not significant.

We next sought to determine the antagonistic efficacy of the conjugative T4SS by revealing the minimal initial donor-to-recipient ratios needed for effective inhibition and susceptible bacterial species. We first employed different ratios (ranging from ~0.3:1 to ~50:1) and performed the experiments against *E. coli* DA32838 lab strain on filter papers as described above. The donor NEB 10-beta *E. coli* strains used for these assays either had T4SS + TrbM or had an empty pCOLADuet-1 vector as a control. Results showed an association between donor concentration and increased lethality, with ~3:1 being the minimal ratio for a ~100-fold reduction in viable recipient cells in comparison to the control treated with the donor containing the empty pCOLADuet-1 vector (Fig. 4a). The reliance on the amount of donor cells present is most likely due to there being a higher likelihood of a recipient cell encountering a donor cell and forming cell-to-cell contact in populations with more donor strains.

Next, we determined the antagonistic efficacy against Gram-negative bacterial species, including *E. coli* New Delhi metallo-β-lactamase-1 (NDM-1)-producing strain (ATCC BAA-2452), *Enterobacter cloacae* NDM-1-producing strain (ATCC BAA-2468), *Klebsiella pneumoniae subsp. pneumoniae* (derived from ATCC BAA-2524), *Pseudomonas aeruginosa* PAO1-LAC (ATCC 47085), and *Pseudomonas putida KT2440* (ATCC 47054) (Fig. 4b). All bacterial species tested except for *P. aeruginosa* were susceptible, as demonstrated by the CFU reduction of the recipients compared to the corresponding controls in which the strains were treated with the donor containing an empty pCOLADuet-1 vector. The inefficacy against *P. aeruginosa* could possibly be explained by the interference caused by its native H1-T6SS, which has been reported to defend and kill donor *E. coli* that attempt to initiate T4SS-dependent conjugative transfer of RP4^83^. Interestingly, despite attacking with its own secretion system, the donor was not significantly inhibited by *P. aeruginosa* PAO1 H1-T6SS as shown by the CFU of the T4SS + TrbM donor being similar to the CFU of the control donor carrying the empty pCOLADuet-1 vector (Supplementary Fig. 15).

To confirm that the H1-T6SS could protect *P. aeruginosa* PAO1 from the T4SS + TrbM donor, we performed experiments in which *Pseudomonas aeruginosa* MPAO1 and *Pseudomonas aeruginosa* MPAO1 *∆retS* (PW9164) were treated with donors carrying either the reconstituted T4SS + TrbM or an empty pCOLADuet-1 vector. We opted for *P. aeruginosa* MPAO1 as the recipient strain of choice due to the lack of commercially available *Pseudomonas aeruginosa* PAO1-LAC mutants^84^. Results from the assays show that, in stark contrast to *Pseudomonas aeruginosa* PAO1-LAC, the WT *Pseudomonas aeruginosa* MPAO1 was susceptible to killing. On the other hand, *Pseudomonas aeruginosa* MPAO1 *∆retS* (PW9164), which has an upregulated H1-T6SS^83^, showed a resistant phenotype similar to *Pseudomonas aeruginosa* PAO1-LAC (Supplementary Fig. 16). The discrepancy between *Pseudomonas aeruginosa* PAO1-LAC and *Pseudomonas aeruginosa* MPAO1 can be attributed genomic differences between the two strains and these genomic differences possibly translating to different regulation and/or expression levels of the H1-T6SS^85^.

Lastly, we asked if the T4SS + TrbM was capable of inhibiting multiple recipients in a mixed-population setting. The lethality of the repurposed RP4-T4SS was further demonstrated by its ability to inhibit a two-strain recipient system composed of *E. coli* DA32838 and *P. putida* KT2440 together (Figs. 4c–4e). This shows that the lethal phenotype is capable of indiscriminate killing and that the presence of more than one strain will not result in the survival of the other strain.

### T4SS originated from conjugative plasmid R388 and also enables interbacterial antagonism

**Fig. 5:**
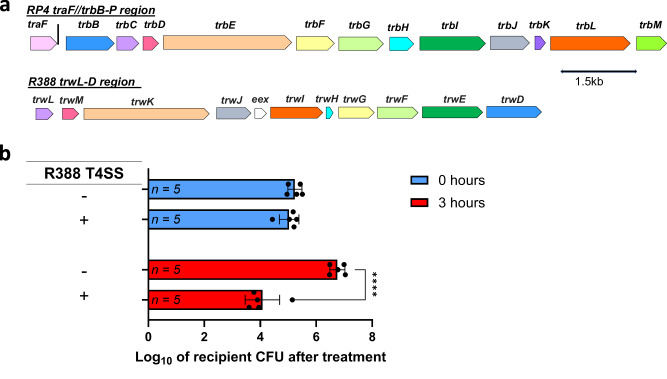
T4SS originated from conjugative plasmid R388 and showed antagonizing capabilities. **a** Schematic depicting homologous T4SS genes between RP4 and R388 obtained from a global pairwise alignment generated by HHpred using default parameters^102^. The amino acid sequences for the RP4 and R388 genes known to be involved in conjugation were used. Matching colors indicate homology was detected (probability value > 87%, E-value < 1.0e−8, and identity value > 15%), and white color indicates a lack of homology. For more details, refer to the supplemental material. **b** Antagonizing capabilities of an *E. coli* NEB® 10-beta donor carrying a reconstituted T4SS-containing operon sourced from R388 against an *E. coli* DA32838 recipient strain. The initial donor CFU was approximately set to the order of 10^7^. The raw CFU of the recipient was first log_10_ transformed and the data shown is the mean of the log_10_ transformed data, with the sample size shown inside the corresponding bar. Error bars represent the standard deviation of the log_10_ transformed data. Two-way RM ANOVA was performed followed by Šídák’s multiple comparisons test. ****P < 0.0001.

Since RP4-derived conjugative T4SS enables interbacterial antagonism, we asked whether the phenotype could also be observed using other conjugative T4SS. We focused on T4SS originated from a well-studied conjugative plasmid belonging to another incompatibility group (i.e., IncW), namely R388, due to its high degree of similarity with T4SS from RP4 (Fig. 5a). We reconstituted the T4SS-containing operon from R388 (Supplementary Data 4) on a cloning vector and showed that an *E. coli* strain harboring this construct was indeed able to antagonize an *E. coli* DA32838 lab strain (Fig. 5b). These results further hint that conjugative plasmids in Gram-negative bacteria may be a hidden source for antagonistic conjugative T4SS.

## Discussion

The work done here showcases direct evidence of a conjugative T4SS encoded by conjugative plasmid RP4, using engineering approaches, being capable of granting a strong antagonistic property to a host *E. coli* strain. The mode of killing is not clear, but speculation leads us to propose that, unlike previously reported examples of bacteria-killing T4SS, the recipient’s death could be the result of catastrophic membrane damage due to excessive attempts at conjugation. Another interesting question that arises is the role of TrbM in enhancing the killing and how it interacts with other components in the T4SS, particularly protein TrbP and TrbO. In addition, with many technologies already having been developed based on cell-to-cell contact, the possibility of harnessing antagonistic conjugative T4SS for a similar purpose seems like an obtainable goal^19–21,86–92^. Future endeavors could be devoted to further characterizing this phenomenon by (1) assessing the type of and the extent of the damage the recipient suffers, (2) obtaining optimal conditions for increased lethality, (3) engineering the system for better efficiency and the ability to overcome the resistance, (4) elucidate the mechanism of killing and (5) uncovering the protein-protein interactions responsible for the phenotype.

More excitingly, the widespread existence of a plethora of conjugative plasmids for both Gram-negative and Gram-positive organisms strongly suggests that there may exist an untapped reservoir of potentially antagonistic conjugative T4SS^93–97^. Even though RP4 and R388 are two well-studied conjugative plasmids, our study is the first demonstration of the antagonistic properties of their encoded conjugative T4SS. In addition, our data showed that the cognate resistance mechanism is specific to the entry exclusion genes carried by the T4SS itself, suggesting that a conjugative T4SS from a different source (non-RP4) could antagonize recipients that are carrying RP4. It is our hope that the work done here sets the foundation for the exploration and development of antimicrobial technologies based on the lethal phenotype showcased in this study.

## Methods

### Reagents & Materials

Platinum™ SuperFi II PCR Master Mix (Invitrogen™) and FastDigest Restriction Enzymes were purchased from Thermo Fisher Scientific. Enzymes for Gibson assembly were from New England BioLabs or Thermo Fisher Scientific. Luria–Bertani (LB) agar and LB broth medium were from BD Difco™. Kanamycin mono sulfate, streptomycin sulfate, chloramphenicol, and tetracycline hydrochloride were from Fisher Bioreagents by Thermo Fisher Scientific; while carbenicillin disodium salt, spectinomycin dihydrochloride pentahydrate, apramycin sulfate [nebramycin II], and Isopropyl-beta-D-thiogalactopyranoside (IPTG) were bought from Research Products International Corp. L-arabinose and trimethoprim were from TCI America^TM^. Chlorophenol red-β-D-galactopyranoside (CPRG) was purchased from Millipore Sigma. Ampicillin sodium salt was obtained from Alfa Aesar. LB agar plates with 6% sucrose without sodium chloride were from Teknova. Whatman™ cellulose acetate membrane filters were purchased from GE Healthcare Sciences (CAT. No. 10404006, 0.45 µm pore size). VWR Porous Adhesive Film for Culture Plates (CAT. No. 60941-086) was purchased from VWR. Cuvettes used for electroporation were Gene Pulser/MicroPulser Electroporation Cuvettes by Bio-Rad (Cat. No. 165-2089). Isolation of plasmid species was done with Zyppy^TM^ Plasmid Miniprep Kit (Zymo Research) and ZR BAC DNA Miniprep Kit (Zymo Research). Mix & Go! *E. coli* Transformation Buffer Set (Zymo Research) was used for making chemically competent *E. coli* cells.

### Bacterial strains & growth conditions

NEB® 10-beta *E. coli* cells were used for cloning. *E. coli* DA32838^53^ was used as the main recipient strain during this study. *E. coli* NEB® 10-beta and *E. coli* DA32838 are intrinsically resistant to streptomycin and chloramphenicol, respectively. Heat shock, electroporation, or bacterial conjugation were used to introduce plasmids into *E. coli* cells. *E. coli NDM-1* (ATCC BAA-2452)*, Enterobacter cloacae* (ATCC BAA-2468)*, Pseudomonas putida* KT2440 (ATCC 47054), and *Pseudomonas aeruginosa* PAO1-LAC (ATCC 47085) were purchased from American Type Culture Collection (ATCC). *Pseudomonas aeruginosa* MPAO1 parent strain and *Pseudomonas aeruginosa* MPAO1 *∆retS* (PW9164 *retS*-A02::ISphoA/hah) were purchased from the Manoil Lab from the University of Washington^84^. *Klebsiella pneumoniae* subsp. *pneumoniae* derived from ATCC BAA-2524 was purchased from Microbiologics. *E. coli* strain BW25113 bearing pIJ790^51,98^, and *E. coli* DY331^99^ were used for λ red recombineering. *E. coli* ET12567 (pUZ8002) was used for obtaining pUZ8002 plasmid^51,100^. *E. coli* XL1-Blue was purchased from Agilent. LB media and 37 °C were used for the propagation of all bacteria, except for *P. aeruginosa* strains, which were cultured in TSB. *P. putida* KT2440 was cultured at 30 °C in LB. Antibiotics concentrations used in the media when appropriate were kanamycin (50 µg/mL), streptomycin (100 µg/mL), chloramphenicol (25 µg/mL), carbenicillin (100 µg/mL), spectinomycin (100 µg/mL), ampicillin (100 µg/mL), apramycin (50 µg/mL), trimethoprim (10 µg/mL).

### Plasmid construction for expression in *E. coli*

Plasmid construction was achieved using standard cloning techniques unless stated otherwise, whereas plasmid design made use of SnapGene software. The oligonucleotide primers used in this study were purchased from MilliporeSigma. Amplified PCR products were recovered using the Zymoclean^TM^ Gel DNA Recovery Kit. Cloning vectors used in this study were pETDuet-1, pCOLADuet-1, or pCDFDuet-1. Plasmid RP4 was used as the model plasmid from which all the studied genes were sourced from. Isolation of plasmid species was done with Zyppy^TM^ Plasmid Miniprep Kit (Zymo Research) and ZR BAC DNA Miniprep Kit (Zymo Research). Plasmids were confirmed by DNA sequencing (Azenta/Genewiz, Plasmidsaurus, or Texas A&M Institute for Genome Sciences and Society).

To construct RP4-GFP (pXJ47), we first sequentially cloned P_J23119_-gfp and aac(3)IV (apramycin-resistant gene) to pCDFDuet-1. A linear DNA fragment of P_J23119_-gfp-*aac(3)IV* flanked by ~40 bp homology to *bla* of RP4 at each end was then amplified from the plasmid by PCR and introduced to RP4 backbone using λ Red homologous recombineering to result in pXJ47. Briefly, *E. coli* strain BW25113 bearing pIJ790 (a plasmid containing λ red genes)^51,100^ and RP4 was grown overnight at 30 °C and at 250 rpm in LB supplemented with kanamycin and chloramphenicol. The resulting strain was then sub-cultured with 10 Mm L-arabinose to induce the expression of λ Red genes at 30 °C to the log phase, washed twice with 10% glycerol, then electroporated with the linear DNA fragment. Once electroporated, the cells were recovered and plated on antibiotic antibiotic-selective plate at 37 °C. Plasmids were mini-prepped using the kit described above and transformed into *E. coli* NEB® 10-beta.

To construct RP4-GFP-ΔoriT (pXJ70), a two-step process of selection and counter-selection was used^98,99^. Briefly, *E. coli* DY331 was used and cultured at 30 °C unless said otherwise. After the induction of λ Red genes in *E. coli* DY331 bearing RP4-GFP (pXJ47) using 42 °C, electrocompetent cells were prepared and electroporated with a linear *cat-sacB* cassette flanked by homology to *traLK-oriT-traJX* at each end to disrupt *traLK-oriT-traJX*. A second round of λ Red homologous recombineering was used to replace *cat-sacB* with a DNA fragment that did not contain the antibiotic resistance gene and *sacB*. The resulting plasmid (pXJ70) was transformed to *E. coli* NEB® 10-beta and confirmed by DNA sequencing.

To inactivate genes on pXJ70, *E. coli* strain BW25113 bearing pIJ790 and pXJ70 was used. The recombineering protocol was similar as used for the construction of pXJ47, except that the DNA fragment to be electroporated into the strain contained *aadA* gene (spectinomycin resistance gene, amplified from pCDFDuet-1) flanked at each end by ~40 bp homology to the gene to be disrupted.

Plasmids, primers, and DNA sequences used are shown in Supplementary Table 1 and Supplementary Data 1–4.

### Antimicrobial assays

The donor strains used in this study were primarily derived from *E. coli* NEB® 10-beta. XL1-1 Blue and DA32838 *E. coli* strains were used as alternative donors for Supplementary Fig. 5. The recipient strains mainly used in this study were *E. coli* DA32838 or its derived strain carrying a pETDuet-1 derived plasmid (pXJZ11, pXJZ45, pXJZ69-82, or pLGV67), or pCOLADuet-1 derived plasmid (pXJZ60 or pLGV96) or a conjugative plasmid (RP4, R388, or R6K). *E. coli NDM-1, E. cloacae, K. pneumoniae*, *P. putida KT2440* and *P. aeruginosa* strains (PAO1-LAC, WT MPAO1, and MPAO1 ∆*retS*) were also used as recipients. Donor and recipient cells were grown overnight at 37 °C and 250 rpm in LB broth (TSB for *P. aeruginosa* strains) supplemented with the appropriate antibiotics. *P. putida* was grown at 30 °C. Specifically, *E. coli* NEB® 10-beta cells without any plasmid were grown in LB supplemented with streptomycin. LB supplemented with kanamycin was used for growing *E. coli* NEB® 10-beta containing RP4, pXJ70, pXJ47, pUZ8002, or any pCOLADuet-1 derived plasmid. Kanamycin was also used for growing *E. coli* XL1-Blue and *E. coli* DA32838 that carried pCOLADuet-1 derived plasmids for Supplementary Fig. 5. Spectinomycin and kanamycin were used for *E. coli* NEB® 10-beta containing plasmid mutants derived from pXJ70; kanamycin and carbenicillin were used when the cells contained both pXJ70-derived plasmid mutants and pETDuet-1 derived plasmids or when the cells contained both pCOLADuet-1 and pETDuet-1 derived plasmids; spectinomycin was used for *E. coli* NEB® 10-beta containing pCDFDuet-1 derived plasmid. *E. coli* DA32838 without plasmid was grown in LB supplemented with chloramphenicol. *E. coli* DA32838 strains containing pETDuet-1 derived plasmids were grown in LB supplemented with chloramphenicol and carbenicillin; kanamycin and chloramphenicol were used for *E. coli* DA32838 strain containing RP4; trimethoprim and carbenicillin were used for *E. coli* DA32838 strains containing R388 and R6K, respectively. *E. coli NDM-1, E. cloacae, K. pneumoniae* and *P. putida* KT2440 were grown in LB supplemented with carbenicillin. *P. aeruginosa* strains were grown in TSB supplemented with ampicillin.

Afterwards, 40 µL of the overnight culture was used to inoculate 4 mL of LB broth with appropriate antibiotics as mentioned above, and the subculture was grown at 37 °C and 250 rpm for two hours. In the case of *Pseudomonas putida*, the subculture was prepared by adding 80 µL of overnight culture into 4 mL of LB with appropriate antibiotics, and the subculture was grown at 30 °C for 2 hours*. Pseudomonas aeruginosa* strains (PAO1-LAC, WT MPAO1, and MPAO1 ∆*retS* PW9164) were not sub-cultured because of their slow growth rate, and its overnight culture was directly used in the following experiment. Next, the donor and recipient were washed twice of pure LB broth to remove any residual antibiotics that may affect downstream experiments.

Antimicrobial assays on membrane filters: Donor and recipient were mixed in different ratios depending on the experiment and 10 µL of each mating mix was spotted on two separate Whatman™ cellulose acetate membrane filters (0.45 µm pore size) placed on LB agar plate. Once dried, the membrane filters were immediately harvested to quantify the initial CFUs of donors and recipients (denoted as 0 hour), while the other filter papers were harvested after the 3-hour treatment at 37 °C. The harvested membrane filters were resuspended in 1 mL of pure LB broth and vigorously vortexed to detach cells from the filter. The resulting suspension was transferred to a 1.5 mL tube and centrifuged at 17,000 *g* for 1 minute. The supernatant was discarded, and the pellet was resuspended in 50 µL of pure LB. Then, 10 µL of the resuspension was used to perform 10-fold serial dilutions. Selective agar media were spotted using 5 µL from the original 50 µL suspension and from the 10-fold serial dilutions. The agar plates were then incubated overnight at 30 °C or 37 °C to allow bacterial growth. LB agar plates supplemented with appropriate antibiotics were used for counting strains based on *E. coli* NEB® 10-beta or recipient strains based on *E. coli* DA32838. LB agar plates supplemented chloramphenicol and kanamycin were used for counting *E. coli* DA32838 transconjugants in Supplementary Fig. 1a. While LB agar plates supplemented chloramphenicol and carbenicillin were used for counting *E. coli* DA32838 transconjugants for Supplementary Table 2. LB agar plates supplemented with carbenicillin were used for counting *E. coli NDM-1, E. cloacae, K. pneumoniae* and *P. putida* KT2440, while LB agar plate with ampicillin was used for counting *P. aeruginosa* strains. Colonies were counted in the dilution that allowed for distinguishable colonies, and the number of dilutions performed that allowed the countable colonies was recorded. The CFUs were determined using the formula: [(number of colonies counted × 10^number of dilutions performed^)/5 µL] × 50 µL.

Mobilizable plasmid transfer assays: *E. coli* NEB® 10-beta donors co-transformed with pLGV146 (plasmid derived from pIB139^101^ that had the apramycin selective marker replaced with carbenicillin resistance marker) and either pXJ70, pXJ47, pUZ8002 or no plasmid were grown overnight in LB supplemented with carbenicillin at 37 °C, while the recipient *E. coli* DA32838 was grown on LB supplemented with chloramphenicol. After subculturing according to the conditions in the Antimicrobial assays section, cells were then prepared in accordance with the Antimicrobial assays on the membrane filters section. Both the donor and recipient CFU were adjusted to approximately 10^6^. Transconjugants were selected using LB plates supplemented with both carbenicillin and chloramphenicol.

Antimicrobial assays when the donor-to-recipient contact was blocked by an additional membrane filter and in liquid conditions: *E. coli* NEB® 10-beta donor containing either an empty vector (pCOLADuet-1) or the reconstituted T4SS + TrbM (pXJZ60) were grown overnight in LB supplemented with kanamycin. The recipient strain, *E. coli* DA32838, was grown in LB supplemented with chloramphenicol. Both strains were grown at 37 °C at 250 rpm, after which the strains were subcultured for 2 hours, and the mating mix was prepared as described above in which the initial donor CFU was set to approximately to the order of 10^7^. For testing under blocked cell-cell contact, 10 µL of donor only was spotted on a piece of filter paper (0.45 µm pore size). Afterward, an additional piece (0.45 µm pore size) was placed directly on top of the donor spot. Then 2.5 µL of the recipient cell was spotted directly on top of where the donor would be. For assaying under liquid conditions, 80 µL of donor was mixed with 20 µL of recipient cells. The resulting 100 µL of mating mix was transferred to a 14 mL culture tube and incubated at 37 °C and 250 rpm for 3 hours. After treatment, pairs in both conditions were harvested by resuspending in 1 mL of LB and spinning down to collect the cell pellet. After which serial dilutions were performed as described previously.

Antimicrobial assays for mixed recipient populations: Overnight cultures of donor *E. coli* NEB® 10-beta containing either an empty vector (pCOLADuet-1) or T4SS + TrbM (pXJZ60), recipient *E. coli* DA32838 containing pCOLADuet-1 and recipient *P. putida* KT2440 were grown on LB supplemented with kanamycin and streptomycin, kanamycin, and chloramphenicol, and carbenicillin respectively. Both *E. coli* strains were incubated overnight at 250 rpm at 37 °C, whereas *P. putida* was incubated at 30 °C and also shaken at 250 rpm. Afterwards, 40 µL of an overnight culture of *E. coli* was used to inoculate 4 mL of LB with appropriate antibiotics, and the subculture was grown for 2 hours at 37 °C at 250 rpm. In the case of *P. putida*, the subculture was prepared by adding 80 µL of overnight culture into 4 mL of LB with appropriate antibiotics, and the subculture was grown at 30 °C for 2 hours. Next, the subcultures were washed with pure LB to remove trace antibiotics, and the cells were resuspended in the following ways: (1) 4 mL of donor *E. coli* NEB® 10-beta were washed and resuspended in 150 µL of LB, and (2) 1 mL of *E. coli* DA32838 and *P. putida* were washed and resuspended in 1 mL of pure LB. Later, the strains were paired up by adding 40 µL of donor strain and 5 µL of each recipient strain (10 µL total) to obtain the mixed recipient population. Then, 10 µL of the mating mix was spotted on filter papers (0.45 µm pore size), with cells being harvested at 0 hours and 3 hours after incubation at 30 °C. Harvesting and serial dilution proceeded as detailed previously.

### Colorimetric CPRG assays

The donor strains used in this study were *E. coli* NEB® 10-beta carrying pXJZ60 or an empty vector pCOLADuet-1. The recipient strain used was *E. coli* DA32838. Donor and recipient cells were grown overnight at 37 °C and at 250 rpm in LB broth supplemented with appropriate antibiotics (kanamycin used for donors, chloramphenicol used for recipients). Afterward, both strains were sub-cultured for two hours in 4 mL of LB broth supplemented with 1 mM IPTG and appropriate antibiotics as mentioned above. 4 mL of donor strains and 1 mL of recipient were washed with 1 mL of pure LB broth twice to remove any residual antibiotics, then resuspended in 150 µL and 1 mL of pure LB broth, respectively. LB agar was supplemented with CPRG to a final concentration of 200 µg/mL. A 96-well plate was used in which the wells were filled with 300 µL of LB agar with CPRG. Any unused wells were instead filled with sterile water to prevent the agar from drying up. Donor and recipient were mixed and 5 µL of the mixture was spotted on the agar in the well; the mating pair ratio used was approximately 1:1, in which the initial CFUs of the recipient were ~10^5^. The controls used were blank media + donor only and blank media + recipient only; as well as wells supplemented with carbenicillin to kill both strains. VWR Porous Adhesive Film was used to seal the plates. Plates were incubated for three hours at 37 °C, followed by 13 hours at 30 °C in a Biotek Epoch 2 microplate reader. 30 °C was used in this assay to avoid background color development on the CPRG agar as mentioned by Paradis-Bleau^80^. The wavelength was set at 574 nm, and readings were taken every thirty minutes for the duration of the incubation period. The readings after three hours at 30 °C were used for plotting in Fig. 3b.

### Contact-dependent killing (CDK) assays

Fluorescently tagged donor (sfGFP) and recipient (dTomato) cells of the same genomic background (*E. coli* NEB® 10-beta) were subcultured in the conditions mentioned in the Bacterial strains & growth conditions section of the supplemental material. Both strains were then washed twice with plain LB to remove residual antibiotics. Strains carrying either pXJZ38 (pCOLADuet-1 + sfGFP) or pXJZ39 (T4SS + TrbM + sfGFP) served as the donors, while the recipient strain carried pETomatola (pETDuet-ColE1::ColA + dTomato). The donor initial CFU was adjusted to ~10^5^ while the initial CFU of the recipient was adjusted to ~10^4^. Afterwards, 3 µL of the donor was spotted on plain LB agar and allowed to dry. Once the donor spot was dry, it was then covered by 10 µL of the recipient resuspension that was spotted immediately adjacent to it and allowed to dry once more at room temperature. After both donor and recipient spots were dried, these were incubated at 37 °C for 3 hours, then 30 °C for 3 hours, and then overnight at 30 °C, with images being taken at the end of each incubation period. The iBright™ FL1500 Imaging System from Thermo Fisher Scientific was used to obtain fluorescent images of donors (Excitation Filter 455 – 485 nm/Emission Filter 508–557 nm) and recipients (Excitation Filter 515 – 545/Emission Filter 568–617 nm) using the Smart Exposure tool to obtain optimal exposure values. The scale bar was added using the (Fiji Is Just) ImageJ (version 1.54 f) software. 100 ×15 mm Petri dishes from VWR (CAT. No. 25384-088) were used for the assays.

### T4SS-LacZ fusion assays

NEB® 10-beta cells containing the LacZ fusions of the T4SS + TrbM, TrbO, and TrbP or their respective controls were subcultured in the conditions mentioned in the Bacterial strains and growth conditions section of the supplemental material. All strains were then washed twice with plain LB to remove residual antibiotics and were then resuspended in 200 µL of LB supplemented with CPRG (200 µg/mL). Next, we aliquoted 100 µL of each strain into wells of Nunc MicroWell 96-Well Optical-Bottom Plates from Thermo Scientific™ that were previously filled with 100 µL of LB supplemented with CPRG (200 µg/mL) and six 2-fold serial dilutions of all the strains were performed. Next, the plate was placed inside the Agilent BioTek Synergy H1 Hybrid Multi-Mode Reader pre-warmed to 37 °C, and absorbance readings at 574 nm were taken every 20 minutes for 16 hours. For Supplementary Fig. 8a, the values corresponding to the readings after 16 hours were used. Due to reading overflow, the values corresponding to the readings of 2 hours were used for Supplementary Fig. 8b.

### Homology detection using HHpred

Amino acid sequences for the RP4 and R388 genes known to be involved in conjugation were subjected to a global pairwise alignment using HHpred^102^. Default parameters were applied, these were: (1) HHblits = >UniRef30 MSA generation method, (2) 3 MSA generation iterations, (3) an E-value cutoff of 1e−3, (4) minimum sequence identity of MSA hits with the query of 0%, (5) a minimum coverage of MSA hits of 20%, and (6) alignment mode of global: realign. Homology was determined using three major factors: (1) A probability value > 87%, (2) E-value < 1.0e−8, and (3) an identity value > 15%.

### Reporting summary

Further information on research design is available in the Nature Portfolio Reporting Summary linked to this article.

### Supplementary information

Supplementary information

Description of Additional Supplementary Files

Supplementary Data 1

Supplementary Data 2

Supplementary Data 3

Supplementary Data 4

Supplementary Data 5

Reporting Summary

## Data Availability

All additional data that support the conclusions from this manuscript are available in the Supplementary Information. Source data for the main figures, oligonucleotide primers used in plasmid construction, DNA fragments used in λ Red homologous recombineering, genes tested for the RP4 and R388 T4SS antimicrobial reconstitutions are supplied as Excel files. The sequences used for the design and construction of the plasmids derived from either RP4 or R388 were based on the available nucleotide sequences found in the National Center for Biotechnology Information (NCBI), under accession numbers BN000925.1 and BR000038.1, respectively. Any additional information is available upon reasonable request to the corresponding author.

## Statistics And Reproducibility

Statistical analyses for all figures were carried out as described in the figure captions. A confidence interval (CI) of 95% was used and statistical significance is denoted as **P* < 0.05; ***P* < 0.01; ****P* < 0.001; *****P* < 0.0001; ns, not significant. All statistical analyses were performed with GraphPad Prism software. All assays were replicated on independent experiments at least three times and technical replicates were avoided.
