# Supplementary material for: Conjugative type IV secretion systems enable bacterial antagonism that operates independently of plasmid transfer
Source: Commun Biol. 2024 Apr 25;7:499. doi: 10.1038/s42003-024-06192-8 (PMC11045733; doi:10.1038/s42003-024-06192-8)
Supplement: Supplementary file 1 — Supplementary information [file 42003_2024_6192_MOESM1_ESM.pdf]

Conjugative Type IV Secretion Systems Enable Bacterial Antagonism that Operates Independently of Plasmid Transfer

Supplementary Information

Supplementary Tables 1 – 2 and **Supplementary Figures 1 – 16** are provided.

Supplementary Table 1: Plasmids used in this study. Antibiotic resistance: kanamycin (Kan), carbenicillin (Carb), apramycin (Apr), spectinomycin (Spec), tetracycline (Tet), trimethoprim (Tmp), streptomycin (Strep).

Name	Based on	Antibiotics used in this study	Purpose
**RP4**	-	Kan	Model plasmid used in study
**RP4-GFP (pXJ47)**	RP4	Kan/Apr	*bla* gene replaced with P_{J23119}-gfp-*aac(3)IV*
**RP4-GFP-*ΔoriT* (pXJ70)**	pXJ47	Kan	Antibiotic marker-free disruption of *traLK-oriT-traJX*.
**pUZ8002**		Kan	RP4 derivative with a deficient *oriT*
**pXJ49**	pCDFDuet-1	Spec/Apr	The plasmid contains a P_{J23119}-gfp-*aac(3)IV* cassette to be amplified for constructing pXJ47
**pXJZ9**	pXJ70	Kan/Spec	*traF* replaced with *aadA* gene
**pXJZ25**	pXJ70	Kan/Spec	*trbB* replaced with *aadA* gene
**pXJZ22**	pXJ70	Kan/Spec	*trbC* replaced with *aadA* gene
**pXJZ26**	pXJ70	Kan/Spec	*trbD* replaced with *aadA* gene
**pLGV13**	pXJ70	Kan/Spec	*trbE* replaced with *aadA* gene

**pXJZ28**	pXJ70	Kan/Spec	*trbF* replaced with *aadA* gene
**pXJZ29**	pXJ70	Kan/Spec	*trbG* replaced with *aadA* gene
**pXJZ30**	pXJ70	Kan/Spec	*trbH* replaced with *aadA* gene
**pXJZ31**	pXJ70	Kan/Spec	*trbI* replaced with *aadA* gene
**pXJZ32**	pXJ70	Kan/Spec	*trbJ* replaced with *aadA* gene
**pXJZ33**	pXJ70	Kan/Spec	*trbK* replaced with *aadA* gene
**pXJZ34**	pXJ70	Kan/Spec	*trbL* replaced with *aadA* gene
**pXJZ35**	pXJ70	Kan/Spec	*trbM* replaced with *aadA* gene
**pXJZ38**	pCOLADuet-1	Kan	Expression of sfGFP in *XmnI* site downstream of kanamycin resistance gene
**pXJZ39**	pXJZ60	Kan	Expression of sfGFP in *SpeI* site downstream of TrbM
**pLGV35**	pXJ70	Kan/Spec	*trbO* replaced with *aadA* gene
**pLGV37**	pXJ70	Kan/Spec	*parE* replaced with *aadA* gene
**pLGV39**	pXJ70	Kan/Spec	*trbN* replaced with *aadA* gene
**pXJZ60**	pCOLADuet-1	Kan	Contains *traF/trbBCDEFGHIJKLM.*<br><br>Main plasmid used in donor strain
**pXJZ61**	pCOLADuet-1	Kan	Contains *traF/trbBCDEFGHIJKL*
**pXJZ11**	pETDuet-1	Carb	Expression of *traF*
**pXJZ69**	pETDuet-1	Carb	Expression of *trbB*

**pXJZ70**	pETDuet-1	Carb	Expression of *trbC*
**pXJZ71**	pETDuet-1	Carb	Expression of t*rbD*
**pXJZ72**	pETDuet-1	Carb	Expression of *trbE*
**pXJZ73**	pETDuet-1	Carb	Expression of *trbF*
**pXJZ74**	pETDuet-1	Carb	Expression of *trbG*
**pXJZ75**	pETDuet-1	Carb	Expression of *trbH*
**pXJZ45**	pETDuet-1	Carb	Expression of *trbI*
**pXJZ76**	pETDuet-1	Carb	Expression of *trbJ*
**pXJZ77**	pETDuet-1	Carb	Expression of *trbL*
**pXJZ78**	pETDuet-1	Carb	Expression of *trbM*
**pXJZ79**	pETDuet-1	Carb	Expression of *trbN*
**pXJZ80**	pETDuet-1	Carb	Expression of *trbJK*
**pXJZ81**	pETDuet-1	Carb	Expression of *trbO*
**pLGV67**	pETDuet-1	Carb	Expression of *trbK*
**pXJZ82**	pETDuet-1	Carb	Expression of *trbP*
**pLGV2**	pCOLADuet-1	Kan	Has *traF/trbFIJKLM*.
**pLGV40**	pLGV2	Kan	*trbCDEGH* inserted between *traF* and *trbF*
**pLGV41**	PLGV2	Kan	*trbBDEGH* inserted between *traF* and *trbF*
**pLGV42**	pLGV2	Kan	*trbBCEGH* inserted between *traF* and *trbF*
**pLGV55**	pLGV2	Kan	*trbBCDGH* inserted between *traF* and *trbF*
**pLGV44**	pLGV2	Kan	*trbBCDEH* inserted between *traF* and *trbF*
**pLGV45**	pLGV2	Kan	*trbBCDEG* inserted between *traF* and *trbF*
**pLGV49**	pCOLADuet-1	Kan	Contains HXF36_RS00010, HXF36_RS00015, HXF36_RS00020 and *trwL-trwD* region of R388

**pLGV59**	pXJ47	Kan/Spec	*trbM* replaced with *aadA* gene
**pLGV61**	pXJZ61	Kan	*trbK* has been cloned out from the T4SS
**pLGV96**	pXJZ60	Kan	*trbJK* have been removed from the reconstituted T4SS + TrbM
**pLGV132**	pETDuet-1	Carb	Expression of *virB11*
**pLGV133**	pETDuet-1	Carb	Expression of *virB5*
**pLGV134**	RP4	Kan	*traK* gene replaced with *aadA* gene
**pLGV145**	pXJZ60	Kan	In-frame fusion of *trbM* with *lacZ* in the *SpeI* site in pXJZ61
**pLGV146**	pIB139	Carb	Replaced apramycin resistance gene with carbenicillin resistance gene. Derived from pIB139[7]
**pLGV150**	pXJZ81	Carb	In-frame fusion of *lacZ* downstream of *trbO*
**pLGV151**	pXJZ82	Carb	In-frame fusion of *lacZ* downstream of *trbP*
**pETomato**	pETDuet-1	Carb	Has dTomato from *E. coli* DA32838 under J29113 promoter
**pETomatola**	pETomato	Carb	ColE1 *oriR* replaced with ColA from pCOLADuet-1
**R388**	-	Tmp	For testing the antimicrobial resistance and source of conjugative T4SS
**R6K**	-	Carb	For testing the antimicrobial resistance

Supplementary Table 2: pUZ8002 and RP4-GFP T4SS are capable of transferring mobilizable plasmid (i.e., pLGV146). Shown is the raw transconjugant CFU after a recipient *E. coli* DA32838 was treated for 3 hours with an *E. coli* NEB® 10-beta donor carrying pLGV146 and co-transformed with either no plasmid, the RP4-GFP-Δ*oriT*, pUZ8002 or RP4-GFP. The mobilizable plasmid used is pLGV146, which is derived from pIB139. The initial CFU for both the donor and recipient strains was approximately 10^6. The transconjugant is defined as the recipient that uptook pLGV146 after 3 hours of treatment.

	**Raw transconjugant CFU after 3 hours of treatment**		
**Donor *E. coli***	**Replicate #1**	**Replicate #2**	**Replicate #3**
WT	0	0	0
RP4-GFP-Δ*oriT*	0	0	0
pUZ8002	2.00E+03	2.00E+02	2.10E+03
RP4-GFP	6.00E+05	8.00E+04	9.00E+05

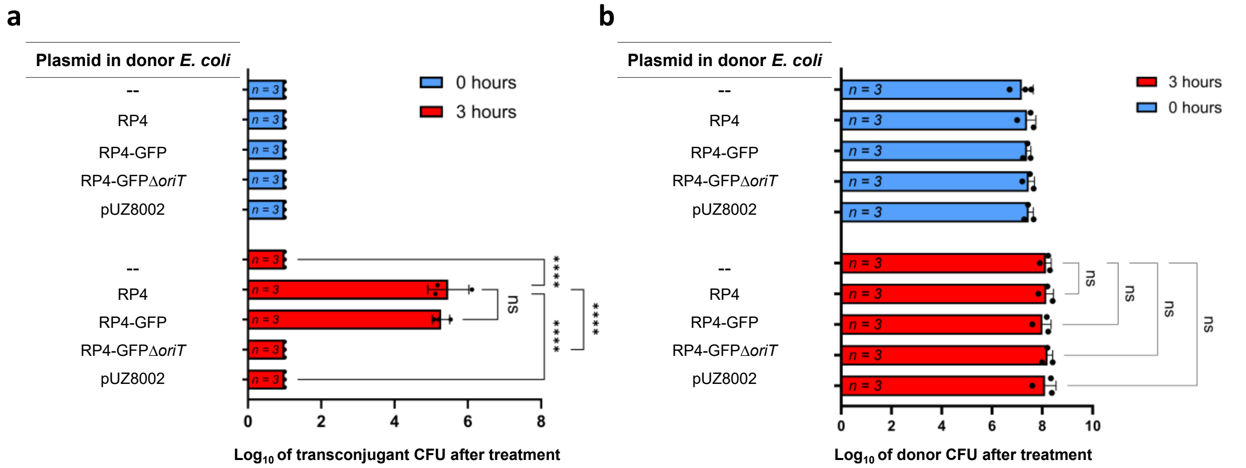

Supplementary Figure 1: RP4-GFP is capable of conjugative transfer and the lethal effect of its RP4-GFPΔ*oriT* derivative is not due to donor cell overgrowth. **(a)** CFU of transconjugant *E. coli* DA32838 cells after 0 and 3 hours of exposure to *E. coli* NEB® 10-beta donor strains carrying the WT RP4, RP4-GFP, RP4-GFP-Δ*oriT* or pUZ8002*.* A WT NEB® 10-beta was used as the control. The raw CFUs of transconjugants were determined from the LB agar plate supplemented with kanamycin and chloramphenicol. No transconjugants were observed when *E. coli* NEB® 10-beta carrying no plasmid, RP4-GFP-Δ*oriT* or pUZ8002 were used as the donors, the CFU detection limit of $[(1x10^0)/5\ \mu L]*50\ \mu L=10$ was used in this case to enable the subsequent log_{10} transformation. **(b)** CFU of different NEB® 10-beta donor bacteria after 0 and after 3 hours of exposure to the *E. coli* DA32838 recipient strain. For both **(a)** and **(b)**, the raw CFU was log_{10} transformed, and the data shown is the mean of the log_{10} transformed data, with the sample size shown inside the corresponding bar. Error bars represent the standard deviation of the log_{10} transformed data. Data shown here are from the same experiments performed for **Figure 1d.** Two-way RM ANOVA were performed followed by Dunnett's multiple comparisons test. **** $P < 0.0001$; ns, not significant.

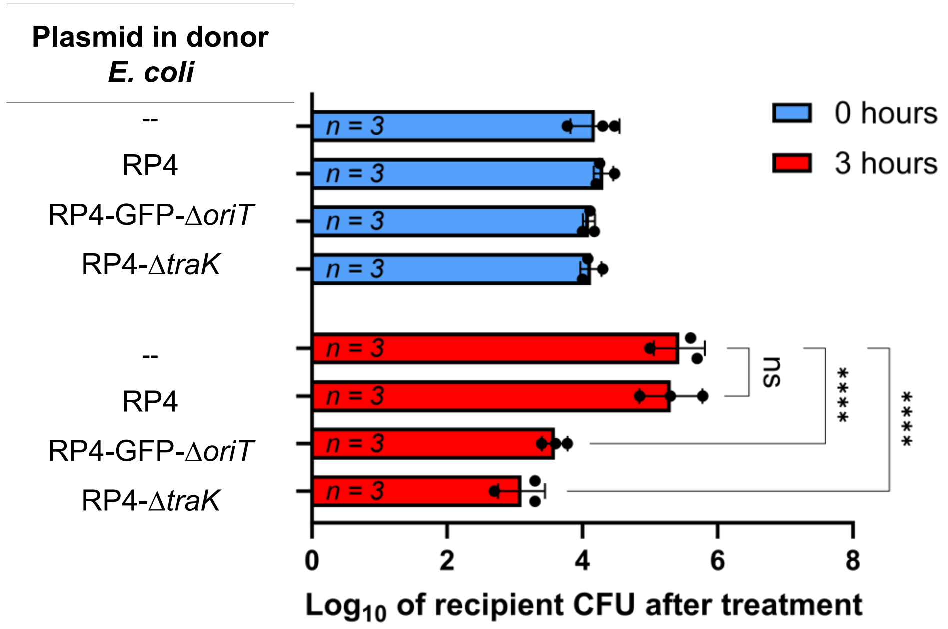

Supplementary Figure 2: Removal of *traK* is sufficient to induce T4SS-mediated antagonism. Shown is the CFU of the recipient *E. coli* DA32838 after 0 and 3 hours of exposure to an *E. coli* NEB® 10-beta donor strain carrying either no plasmid, WT RP4, RP4-GFP-Δ*oriT or* RP4-Δ*traK.* The initial CFU of the donor was approximately set to the order of 10^6. The raw CFU of the recipient was first $\log_{10}$ transformed and the data shown is the mean of the $\log_{10}$ transformed data, with the sample size shown inside the corresponding bar. Error bars represent the standard deviation of the $\log_{10}$ transformed data. Two-way RM ANOVA were performed followed by Dunnett's multiple comparisons test. **** $P < 0.0001$; ns, not significant.

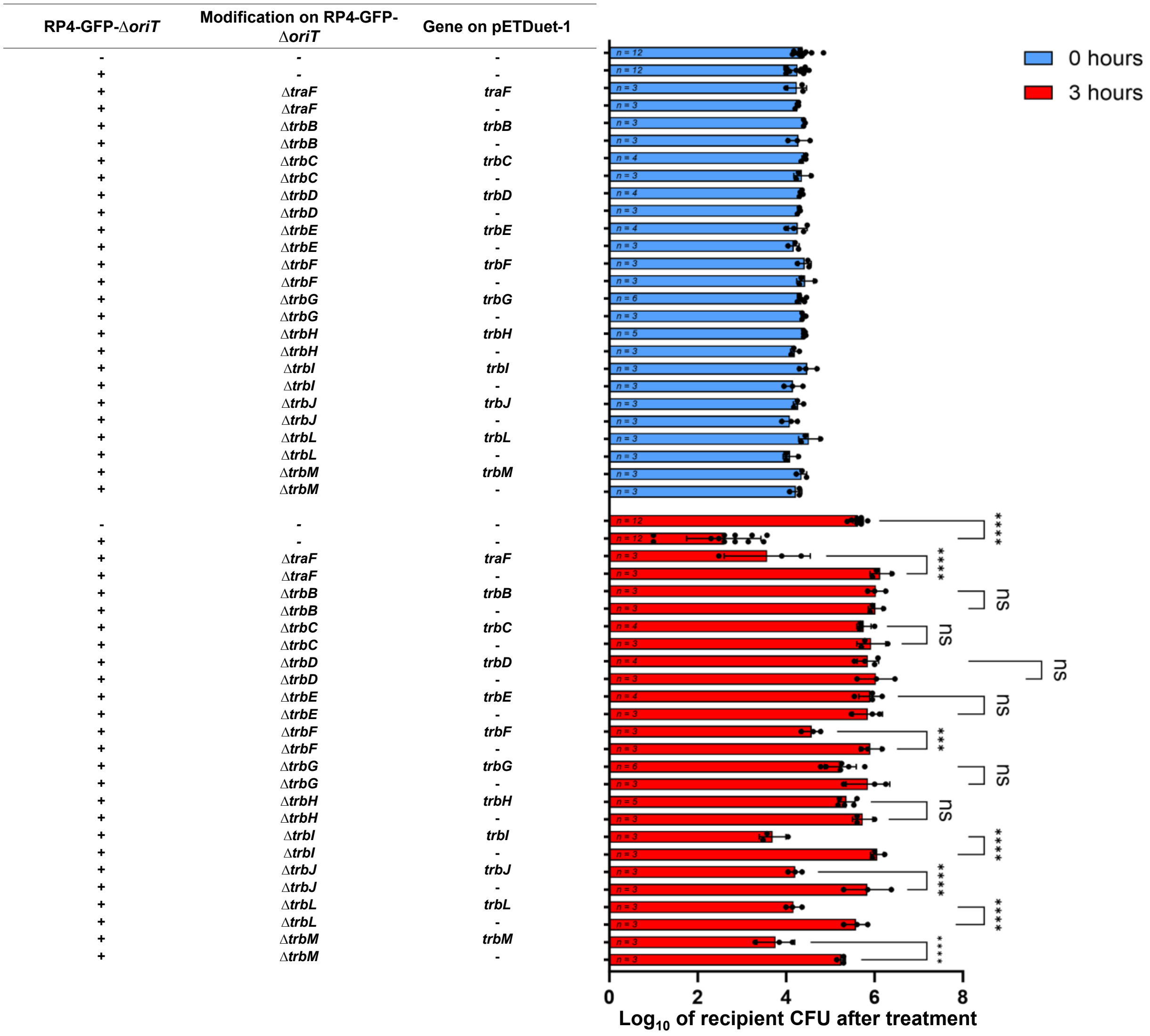

Supplementary Figure 3: Complementation of the RP4-GFP-Δ*oriT* T4SS mutants. Shown is the CFU of recipient *E. coli* DA32838 after 0 and 3 hours of exposure to *E. coli* NEB® 10-beta donors equipped with RP4-GFP-Δ*oriT* knockout mutants complemented with the appropriate WT gene. NEB® 10-beta donors with RP4-GFP-Δ*oriT* or no plasmid were used as controls. Whenever no colonies of recipients were observed, the CFU detection limit of $[(1\times10^0)/5\ \mu L]*50\ \mu L=10$ was used to enable the subsequent $\log_{10}$ transformation. The initial donor CFU was approximately set to the order of 10^7. The raw CFU of the recipient was first $\log_{10}$ transformed and the data shown is the mean of the $\log_{10}$ transformed data, with the sample size shown inside the corresponding bar. Error bars represent the standard deviation of the $\log_{10}$ transformed data. Two-way RM ANOVA were performed followed by Tukey's multiple comparisons test. *** $P < 0.001$; **** $P < 0.0001$; ns, not significant.

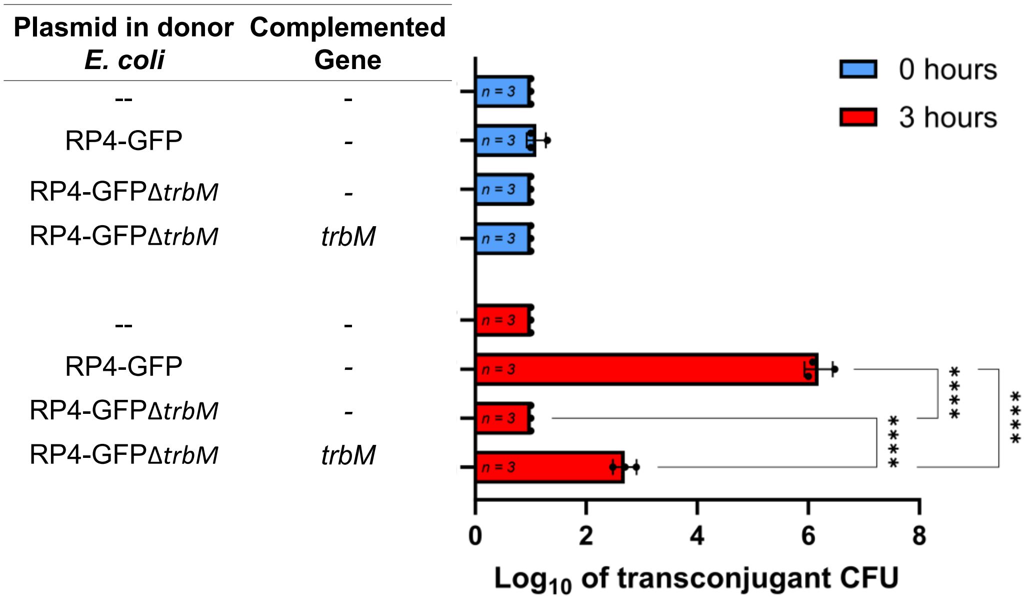

Supplementary Figure 4: Removing *trbM* abolishes conjugation. Shown is the CFU of the *E. coli* DA32838 transconjugants after 0- and 3-hours of exposure to an *E. coli* NEB® 10-beta donor strain carrying either no plasmid, RP4-GFP, or RP4-GFP-Δ*trbM* mutants that were complemented with either an empty pETDuet-1 vector or the WT *trbM* gene. The initial CFU of the donor was approximately set to the order of 10^7. The raw CFU of the transconjugants was first $\log_{10}$ transformed and the data shown is the mean of the $\log_{10}$ transformed data, with the sample size shown inside the corresponding bar. Error bars represent the standard deviation of the $\log_{10}$ transformed data. Two-way RM ANOVA were performed followed by Tukey's multiple comparisons test. **** $P < 0.0001$.

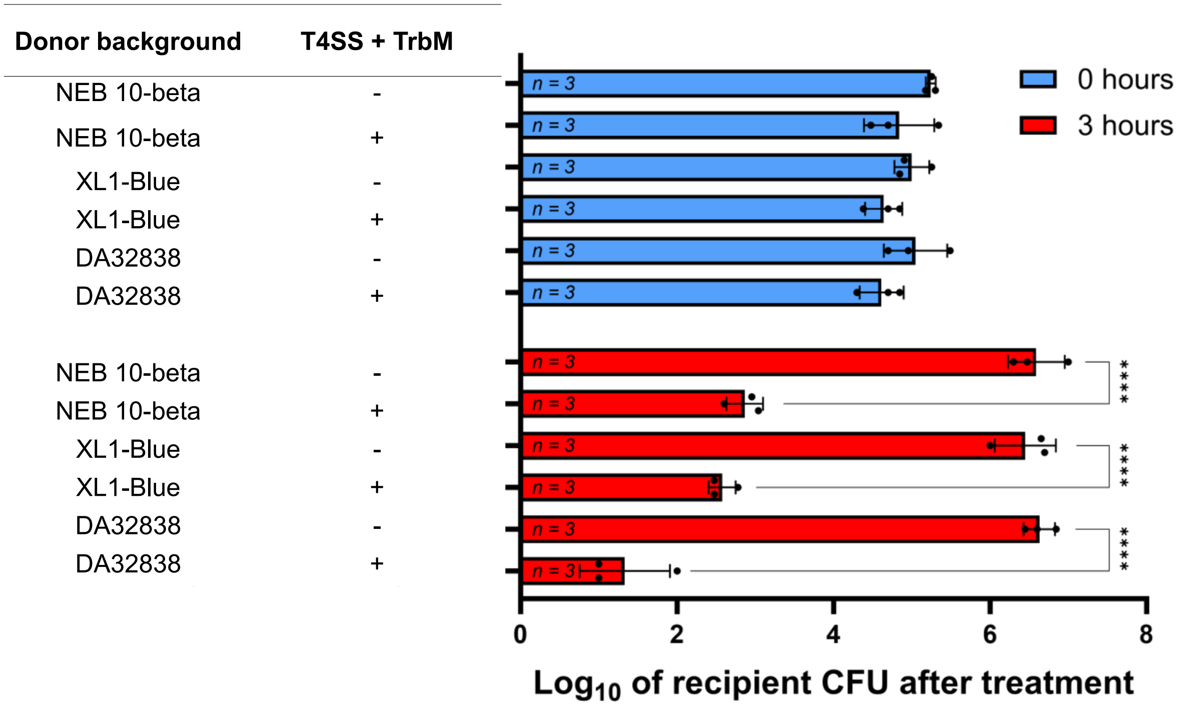

Supplementary Figure 5: The lethal phenotype is not specific to a single *E. coli* strain. Shown is the CFU of the recipient *E. coli* strain after 0 and 3 hours of exposure to either NEB® 10-beta, DA32838, or XL1-Blue *E. coli* donor strains carrying either the T4SS + TrbM or the empty pCOLADuet-1 vector as a control. The initial donor CFU was approximately set to the order of 10^6. The raw CFU of the recipient was first $\log_{10}$ transformed, and the data shown is the mean of the $\log_{10}$ transformed data, with the sample size shown inside the corresponding bar. Error bars represent the standard deviation of the $\log_{10}$ transformed data. Two-way RM ANOVA were performed followed by Tukey's multiple comparisons test. **** $P < 0.0001$.

traF/trbBCDEFGHIJKLM	Modification on traF/trbBCDEFGHIJKLM	Gene on pETDuet-1
+	-	-
-	-	-
+	ΔtrbB	trbB
+	ΔtrbB	-
+	ΔtrbC	trbC
+	ΔtrbC	-
+	ΔtrbD	trbD
+	ΔtrbD	-
+	ΔtrbE	trbE
+	ΔtrbE	-
+	ΔtrbG	trbG
+	ΔtrbG	-
+	ΔtrbH	trbH
+	ΔtrbH	-
+	-	-
-	-	-
+	ΔtrbB	trbB
+	ΔtrbB	-
+	ΔtrbC	trbC
+	ΔtrbC	-
+	ΔtrbD	trbD
+	ΔtrbD	-
+	ΔtrbE	trbE
+	ΔtrbE	-
+	ΔtrbG	trbG
+	ΔtrbG	-
+	ΔtrbH	trbH
+	ΔtrbH	-

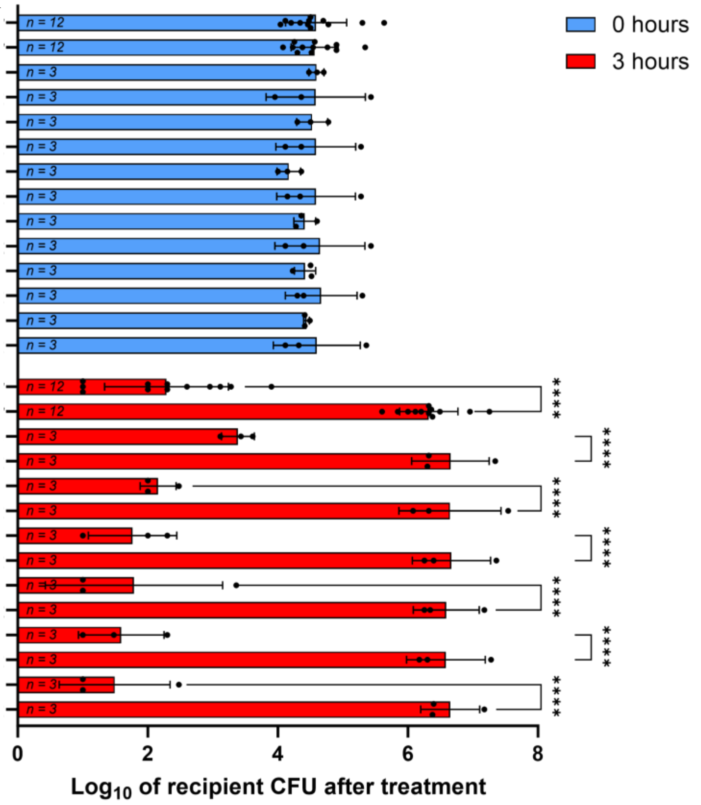

Supplementary Figure 6: Complementation of *trbBCDEGH* mutants in the reconstituted T4SS system. Shown is the CFU of recipient *E. coli* DA32838 after 0 and 3 hours of exposure to the *E. coli* NEB® 10-beta donor strains containing the reconstituted knockouts of *trbBCDEGH* complemented with either the appropriate WT gene or an empty pETDuet-1 vector as a control. NEB® 10-beta donors carrying pCOLADuet-1 and pETDuet-1 were used as an additional control. Data was plotted as the $\log_{10}$ of the colony forming units. Whenever no colonies of recipients were observed, the CFU detection limit of $[(1x10^0)/ 5\ \mu L]*50\ \mu L=10$ was used to enable the subsequent $\log_{10}$ transformation. The initial donor CFU was approximately set to the order of 10^6. The raw CFU of the recipients was first $\log_{10}$ transformed, and the data shown is the mean of the $\log_{10}$ transformed data, with each sample size shown inside the corresponding bar. Error bars represent the standard deviation of the $\log_{10}$ transformed data. Two-way RM ANOVA were performed followed by Tukey's multiple comparisons test. **** $P < 0.0001$.

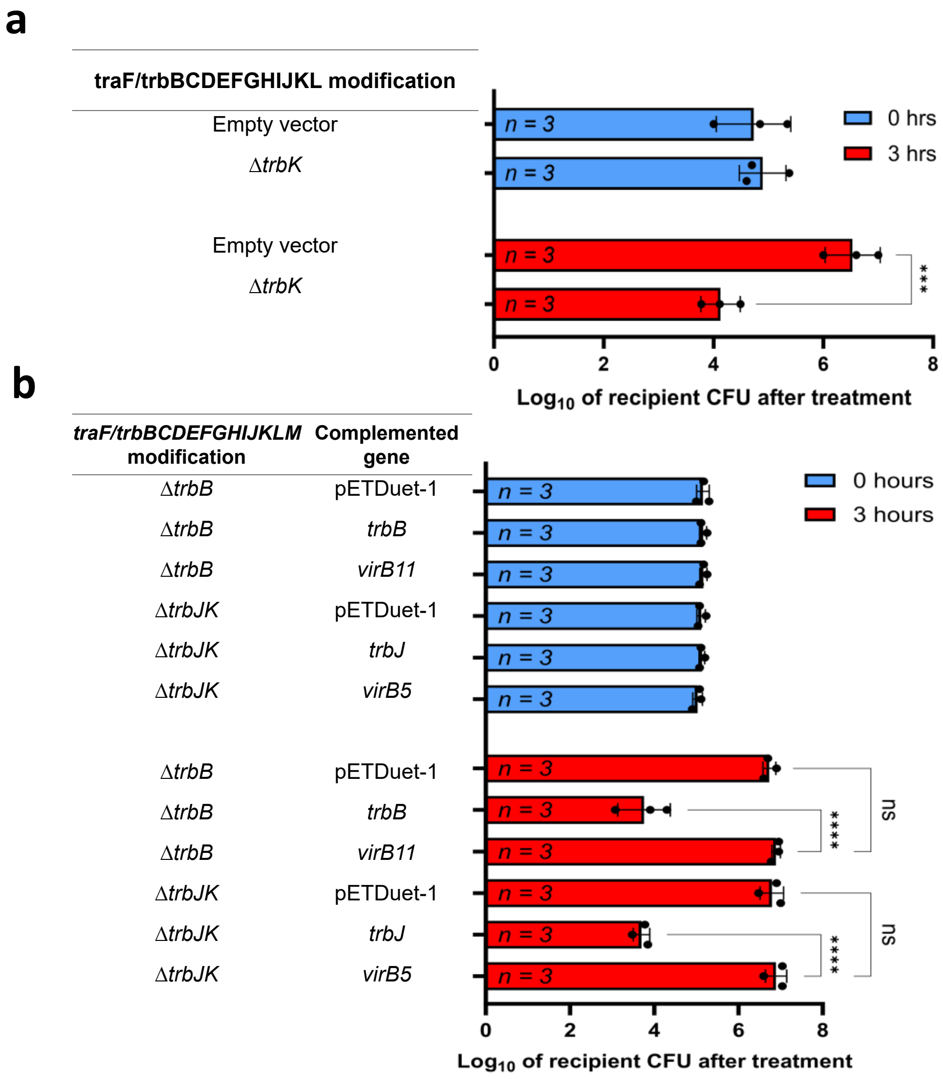

Supplementary Figure 7: The lethal phenotype of the reconstituted RP4-T4SS does not require *trbK* and knockouts of essential components cannot be complemented by their respective *virB/D4* homologue from the *Agrobacterium tumefaciens* T4SS. **(7a)** CFU of a recipient *E. coli* DA32838 strain after 0 and 3 hours of exposure to to *E. coli* NEB® 10-beta donor strains carrying either a *trbK* knockout of the reconstituted T4SS (pXJZ61) or an empty pCOLADuet-1 vector control. **(7b)** CFU of a recipient *E. coli* DA32838 strain after 0 and 3 hours of exposure to to *E. coli* NEB® 10-beta donor strains carrying the Δ*trbB* or Δ*trbJK* knockouts derived from the T4SS + TrbM co-transformed with either an empty pETDuet-1 vector, the WT *trbB/trbJ* gene or the *virB11/virB5* homologue from the *A. tumefaciens* T4SS. For both **(a)** and **(b)**, the initial donor CFU was approximately set to the order of 10^6. The raw CFU of the recipient was first $\log_{10}$ transformed and the data shown is the mean of the $\log_{10}$ transformed data, with the sample size shown inside the corresponding bar. Error bars represent the standard deviation of the $\log_{10}$ transformed data**.** For **(a)**, two-way RM ANOVA were performed followed by Šídák's multiple comparisons test. For **(b)**, two-way RM ANOVA were performed followed by Tukey's multiple comparisons test. *** P<0.001; **** P < 0.0001; ns, not significant.

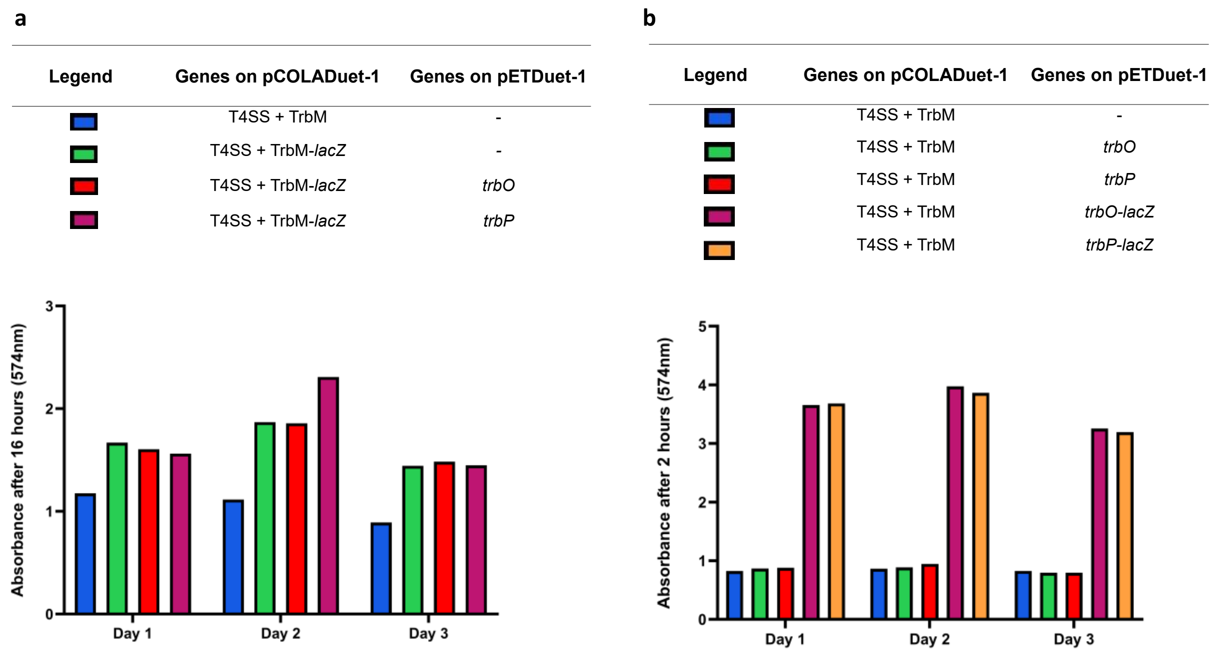

Supplementary Figure 8: Confirmation of T4SS gene expression using LacZ fusions and CPRG hydrolysis. **(a)** LacZ was fused in-frame to TrbM at the end of the T4SS + TrbM construct (pXJZ60) and co-expressed with either an empty pETDuet-1 vector, *trbP* or *trbO* in the same cell. A T4SS + TrbM construct with no fusion co-transformed with an empty pETDuet-1 vector was used as a control. **(b)** LacZ was fused in-frame to either *trbO* or *trbP* and co-expressed with the T4SS + TrbM construct in the same cell. A T4SS + TrbM construct with no fusion co-transformed with an empty pETDuet-1 vector was used as a control. *E. coli* NEB® 10-beta served as the host strain. For both figures, hydrolysis of CPRG (200µg/mL) was detected via absorbance at 574nm. Independent replicates were performed on three different days, with each bar representing a single replicate.

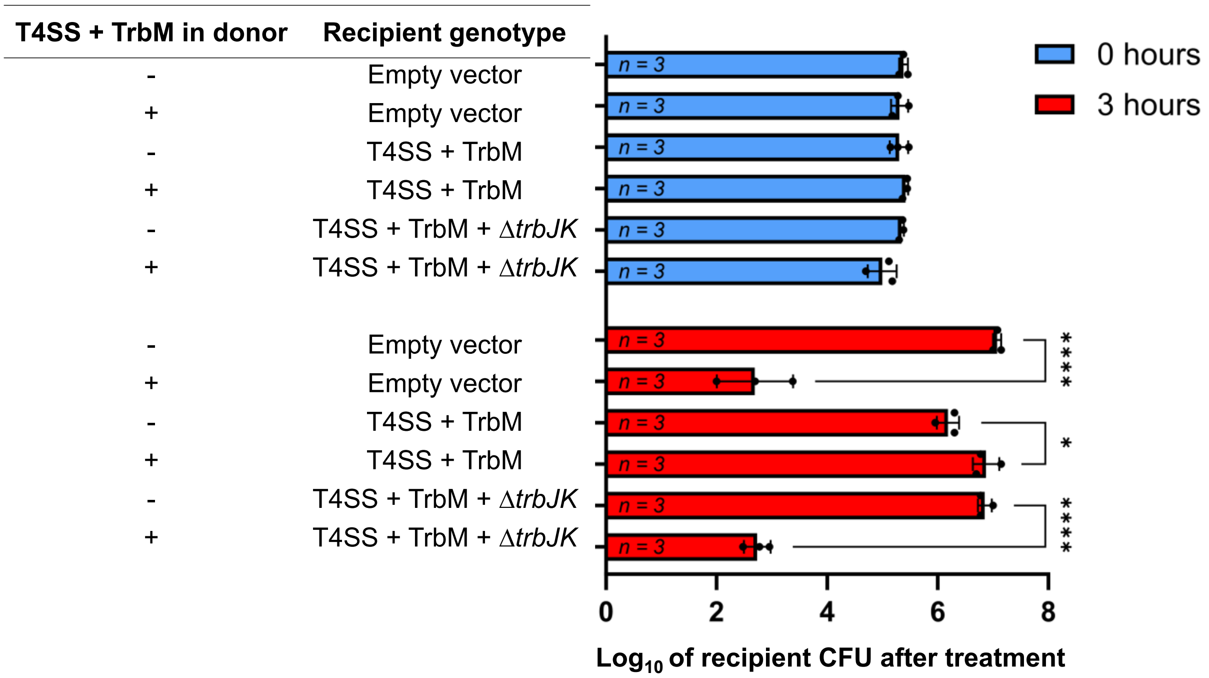

Supplementary Figure 9: Knockouts of *trbJK* in the reconstituted T4SS + TrbM lose resistance. Shown is the CFU of *E. coli* DA32838 recipients carrying either an empty pCOLADuet-1 vector, the reconstituted T4SS or the reconstituted T4SS with *trbJK* knocked out after 0 and 3 hours of exposure to *E. coli* NEB® 10-beta donors equipped with either the reconstituted T4SS + TrbM or an empty pCOLADuet-1 vector control. The initial donor CFU was approximately set to the order of 10^7. The raw CFU of the recipients was first $\log_{10}$ transformed and the data shown is the mean of the $\log_{10}$ transformed data, with the sample size shown inside the corresponding bar. Error bars represent the standard deviation of the $\log_{10}$ transformed data**.** Two-way RM ANOVA were performed followed by Tukey's multiple comparisons test. * $P < 0.05$; **** $P < 0.0001$; ns, not significant.

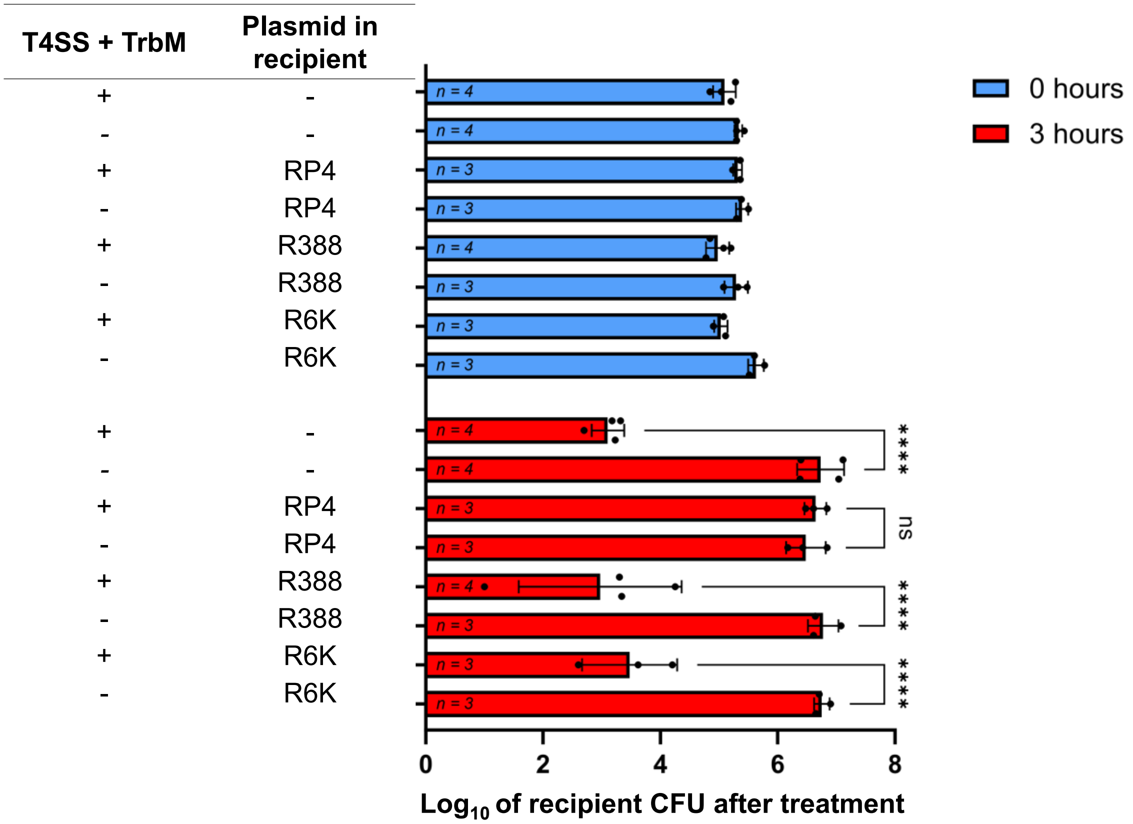

Supplementary Figure 10: Other entry exclusion systems do not confer resistance towards the reconstituted RP4-T4SS. Shown is the CFU of recipient cells *E. coli* DA32838 bearing different conjugative plasmids after 0 and 3 hours of exposure to a donor *E. coli* NEB® 10-beta carrying an empty pCOLADuet-1 or the reconstituted T4SS +TrbM. Plasmidless recipient and donor with an empty pCOLADuet-1 vector were used as controls. The initial donor CFU was approximately set to the order of 10^7. The raw CFU of the recipients was first $\log_{10}$ transformed and the data shown is the mean of the $\log_{10}$ transformed data, with the sample size shown inside the corresponding bar. Error bars represent the standard deviation of the $\log_{10}$ transformed data**.** Two-way RM ANOVA were performed followed by Tukey's multiple comparisons test. **** $P < 0.0001$; ns, not significant.

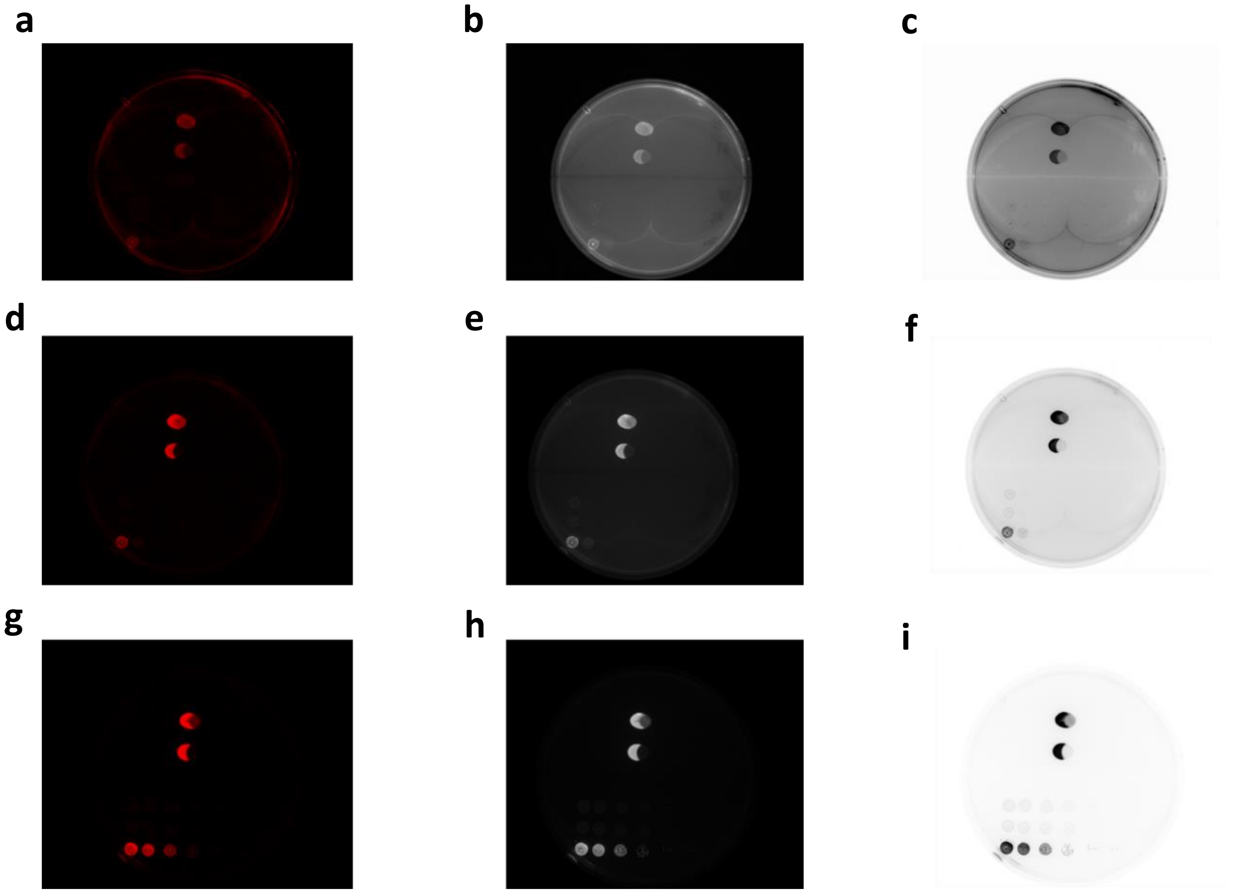

Supplementary Figure 11: Unedited images for the contact dependent killing (CDK) assays performed for **Figure 3e** showing the recipient *E. coli* NEB® 10-beta carrying plasmid pETomatola expressing dTomato. Images were taken using the iBright™ FL1500 Imaging System from Thermo Fisher Scientific using the excitation filter 515 – 545nm and emission filter 568 – 617nm. Shown is the plate after 3 hours of incubation at 37ºC **(a – c)**, followed by 3 hours of incubation at 30ºC **(d – f)** and overnight incubation at 30ºC **(g – i)**. Images are shown in composite **(a, d & g)**, raw image **(b, e & h)** and fluorescent signal **(c, f & i)** formats. The recipient exposed to an *E. coli* NEB® 10-beta donor carrying the control plasmid (pXJZ38) corresponds to the top spot on the plate, while the recipient exposed to an *E. coli* NEB® 10-beta donor carrying the T4SS + TrbM construct (pXJZ39) corresponds to the spot below it. Shown at the bottom of the plate is the 10-fold serial dilution of the recipient *E. coli* NEB® 10-beta carrying plasmid pETomatola expressing dTomato collected before the incubation. Images were taken on 100 x 15 mm Petri dishes from VWR (CAT. No. 25384-088).

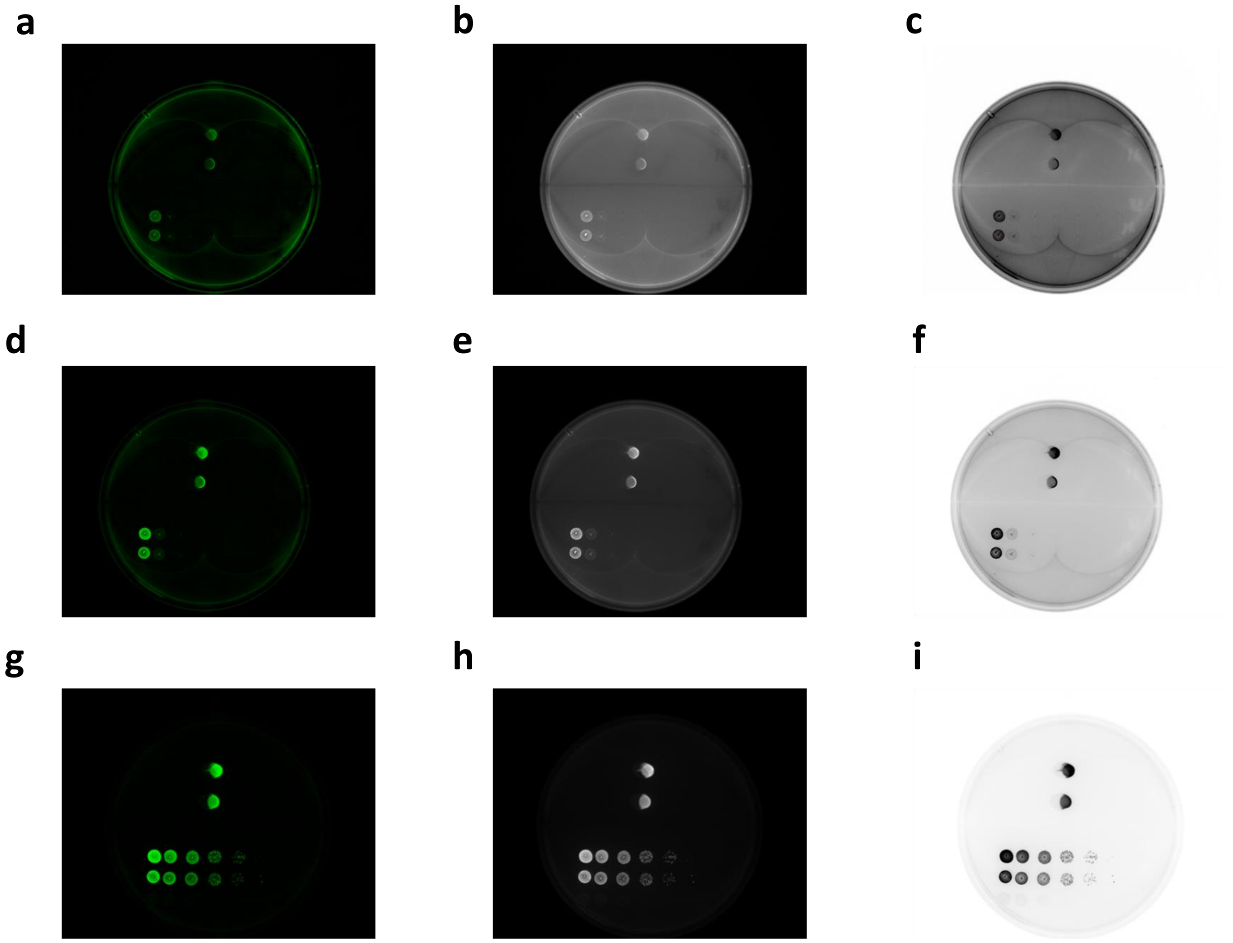

Supplementary Figure 12: Unedited images for the contact dependent killing (CDK) assays performed for **Figure 3e** showing the donor *E. coli* NEB® 10-beta expressing GFP. Images were taken using the iBright™ FL1500 Imaging System from Thermo Fisher Scientific using the excitation filter 455 – 485nm and emission filter 508 – 557nm. Shown is the plate after 3 hours of incubation at 37ºC **(a – c)**, followed by 3 hours of incubation at 30ºC **(d – f)** and overnight incubation at 30ºC **(g – i)**. Images are shown in composite **(a, d & g)**, raw image **(b, e & h)** and fluorescent signal **(c, f & i)** formats. The donor *E. coli* NEB® 10-beta carrying the control plasmid (pXJZ38) corresponds to the top spot on the plate, while the donor *E. coli* NEB® 10-beta carrying the T4SS + TrbM construct (pXJZ39) corresponds to the spot below it. Shown at the bottom of the plate are the 10-fold serial dilutions of the *E. coli* NEB® 10-beta donor carrying either the control plasmid (top dilution) or the T4SS + TrbM construct (bottom dilution) collected before the incubation. Images were taken on 100 x 15 mm Petri dishes from VWR (CAT. No. 25384-088).

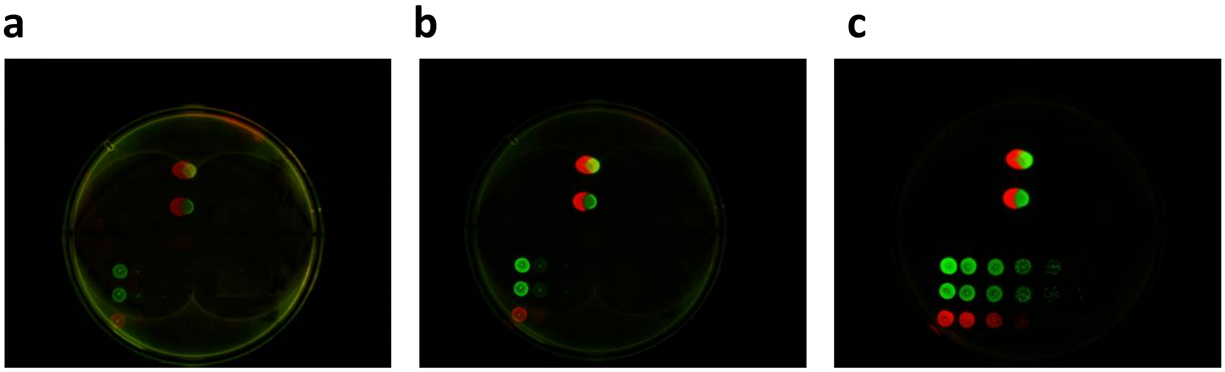

Supplementary Figure 13: Unedited images for the contact dependent killing (CDK) assays performed for **Figure 3e** showing the both the recipient *E. coli* NEB® 10-beta expressing dTomato and the donor *E. coli* NEB® 10-beta expressing GFP. Images were taken using the iBright™ FL1500 Imaging System from Thermo Fisher Scientific using the excitation filter 515 – 545nm and emission filter 568 – 617nm for imaging the donor and the excitation filter 455 – 485nm and emission filter 508 – 557nm for imaging the recipient. Shown is the plate after 3 hours of incubation at 37ºC **(a)**, followed by 3 hours of incubation at 30ºC **(b)** and overnight incubation at 30 ºC **(c)**. The recipient exposed to the donor *E. coli* NEB® 10-beta carrying the control plasmid (pXJZ38) corresponds to the top spot on the plate, while the recipient exposed to the donor *E. coli* NEB® 10-beta carrying the T4SS + TrbM construct (pXJZ39) corresponds to the spot below it. Shown at the bottom of the plate are the 10-fold serial dilutions corresponding to the donor carrying the control plasmid (top dilution), the donor carrying the T4SS + TrbM construct (middle dilution) or the recipient carrying pETomatola (bottom dilution) collected before the incubation. Images were taken on 100 x 15 mm Petri dishes from VWR (CAT. No. 25384-088).

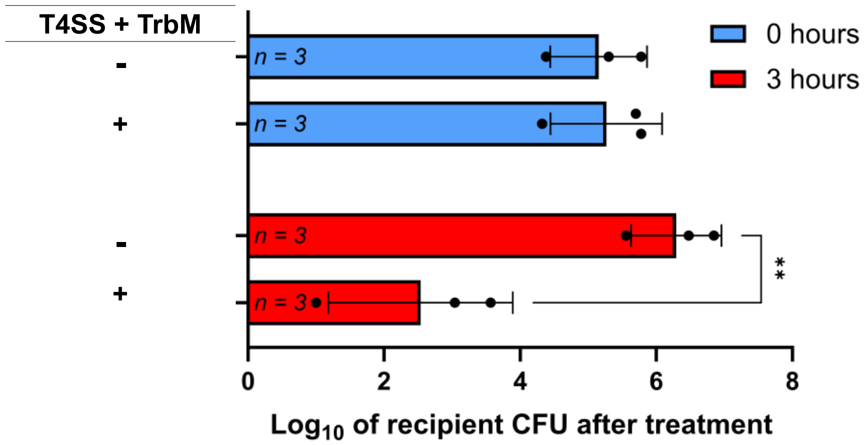

Supplementary Figure 14: Host-secreted molecules do not contribute to the lethal effect. Shown is the CFU of recipient *E. coli* NEB® 10-beta after 0 and 3 hours of exposure to *E. coli* NEB® 10-beta donors carrying the reconstituted T4SS + TrbM or an empty pCOLADuet-1 vector as a control. Recipient strains were transformed pETDuet-1 containing carbenicillin resistance. The initial donor CFU was approximately set to the order of 10^6. The raw CFU of the recipients was first $\log_{10}$ transformed and the data shown is the mean of the $\log_{10}$ transformed data, with the sample size shown inside the corresponding bar. Error bars represent the standard deviation of the $\log_{10}$ transformed data. Two-way RM ANOVA were performed followed by Šídák's multiple comparisons test. ** $P < 0.01$.

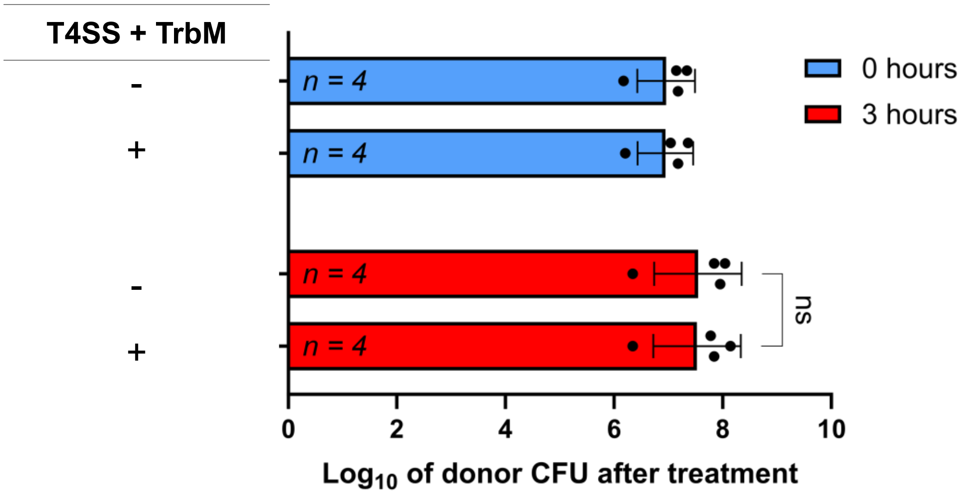

Supplementary Figure 15: Donors expressing the lethal reconstituted RP4-T4SS are not significantly inhibited by *Pseudomonas aeruginosa* PAO1-LAC H1-T6SS. Shown is the colony forming unit (CFU) of the NEB® 10-beta donor bacteria after 0 and 3 hours of exposure to a *Pseudomonas aeruginosa* PAO1-LAC recipient strain. The raw CFU of the donors was first $\log_{10}$ transformed, and the data shown is the mean of the $\log_{10}$ transformed data, with the sample size shown inside the corresponding bar. Error bars represent the standard deviation of the $\log_{10}$ transformed data. Data shown here are from the same experiments performed for **Figure 4b**. Two-way RM ANOVA were performed followed by Šídák's multiple comparisons test. ns, not significant.

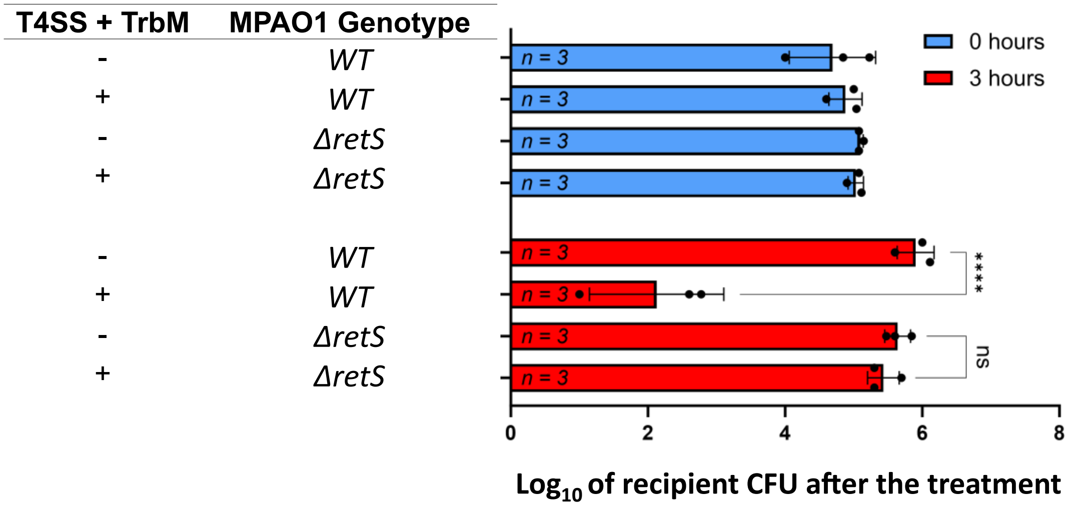

Supplementary Figure 16: The reconstituted lethal RP4-T4SS can eliminate *Pseudomonas aeruginosa* MPAO1 but not its Δ*rets* mutant. Shown is the CFU of a WT *Pseudomonas aeruginosa* MPAO1 and *Pseudomonas aeruginosa* MPAO1 Δ*rets* (PW9164) recipients. *E. coli* NEB® 10-beta carrying either the reconstituted T4SS + TrbM or an empty pCOLADuet-1 vector control served as the donor strains. The initial donor CFU was approximately set to the order of 10^6. The raw CFU of the recipients was first $\log_{10}$ transformed, and the data shown is the mean of the $\log_{10}$ transformed data, with the sample size shown inside the corresponding bar. Error bars represent the standard deviation of the log10 transformed data. Two-way RM ANOVA were performed followed by Tukey's multiple comparisons test. ****$P < 0.0001$; ns, not significant.
