# Supplementary material for: Conjugative type IV secretion systems enable bacterial antagonism that operates independently of plasmid transfer
Source: Commun Biol. 2024 Apr 25;7:499. doi: 10.1038/s42003-024-06192-8 (PMC11045733; doi:10.1038/s42003-024-06192-8)
Supplement: Supplementary file 2 — Description of Additional Supplementary Files [file 42003_2024_6192_MOESM2_ESM.pdf]

Description of Additional Supplementary Files

File name: Supplementary Data 1

Description: Oligonucleotide primers used in plasmid construction

File name: Supplementary Data 2

Description: DNA fragments used in λ Red homologous recombineering and in the construction of pLGV132 and pLGV133.

File name: Supplementary Data 3

Description: Genes tested during RP4 T4SS antimicrobial reconstitution.

File name: Supplementary Data 4

Description: Genes used for the R388 T4SS antimicrobial reconstitution

File name: Supplementary Data 5

Description: Source data for Figures 1 – 5.
